# Visualizing the failure of solid electrolyte under GPa-level interface stress induced by lithium eruption

**DOI:** 10.1038/s41467-022-32732-z

**Published:** 2022-08-27

**Authors:** Haowen Gao, Xin Ai, Hongchun Wang, Wangqin Li, Ping Wei, Yong Cheng, Siwei Gui, Hui Yang, Yong Yang, Ming-Sheng Wang

**Affiliations:** 1 State Key Laboratory of Physical Chemistry of Solid Surfaces, College of Materials, Xiamen University, 361005 Xiamen, China; 2 State Key Laboratory of Material Processing and Die & Mould Technology, Department of Mechanics, School of Aerospace Engineering, Huazhong University of Science and Technology, Wuhan, 430074 Hubei China; 3 State Key Laboratory of Physical Chemistry of Solid Surfaces, College of Chemistry and Chemical Engineering, College of Energy, Xiamen University, Xiamen, 361005 Fujian China

**Keywords:** Batteries, Nanoparticles, Batteries

## Abstract

Solid electrolytes hold the promise for enabling high-performance lithium (Li) metal batteries, but suffer from Li-filament penetration issues. The mechanism of this rate-dependent failure, especially the impact of the electrochemo-mechanical attack from Li deposition, remains elusive. Herein, we reveal the Li deposition dynamics and associated failure mechanism of solid electrolyte by visualizing the Li|Li_7_La_3_Zr_2_O_12_ (LLZO) interface evolution via in situ transmission electron microscopy (TEM). Under a strong mechanical constraint and low charging rate, the Li-deposition-induced stress enables the single-crystal Li to laterally expand on LLZO. However, upon Li “eruption”, the rapidly built-up local stress, reaching at least GPa level, can even crack single-crystal LLZO particles without apparent defects. In comparison, Li vertical growth by weakening the mechanical constraint can boost the local current density up to A·cm^−2^ level without damaging LLZO. Our results demonstrate that the crack initiation at the Li|LLZO interface depends strongly on not only the local current density but also the way and efficiency of mass/stress release. Finally, potential strategies enabling fast Li transport and stress relaxation at the interface are proposed for promoting the rate capability of solid electrolytes.

## Abstract (web summary)

The mechanism of lithium dendrites penetrating solid electrolytes remains elusive. Herein, the authors reveal the Li deposition dynamics and the associated failure mechanism of solid electrolyte by visualizing the Li|LLZO interface evolution via in situ transmission electron microscopy.

## Introduction

Solid electrolytes (SEs) with competitive ionic conductivity are widely considered as a promising enabler of lithium (Li) metal anodes for next-generation high-energy-density Li battery systems^1–3^. However, the practical use of SEs in solid state batteries (SSBs) has been severely hindered by Li dendrite penetration and the associated low critical current density (CCD). Even the high-stiffness ceramic SEs, such as Li_7_La_3_Zr_2_O_12_ (LLZO), cannot suppress Li penetration above the CCD, which is usually below a few mA·cm^−2^ ^4–6^.

Several mechanisms have been proposed to address the complexity of dendrite growth in various SE systems^4–15^. It is now commonly accepted that Li penetration can be initiated at the Li|SE interfaces or within the SEs. In the former scenario, the pre-existing surface flaws, e.g., grain boundaries, scratch, or pits, can induce current focusing and stress concentration, resulting in the subsequent SE fracture and Li penetration^2^. Specifically, Porz et al. predicted a critical flaw size for crack extension at a given stress, which has been widely used to describe the crack initiation at the SE surface^10^. Recent studies on the elasto-plastic response of Li whiskers revealed the high yield strength of nanosized Li (up to 244 MPa), which indicates a plastic limitation of Porz et al’s model^16^. Take LLZO for example, this limitation suggests that if the surface flaw size is reduced below a few microns, the LLZO can resist the electrochemo-mechanical attack from Li due to the stress release by Li plastic yielding (and by the appreciable creep of Li at room temperature as well). However, several works showed that even for the single-crystal LLZO with only submicron or smaller surface flaws, Li penetration was still unavoidable at high charging rates^10,17,18^. For instance, Li penetration can be initiated at the surface defects of 0.4 μm, which corresponds to a hydrostatic pressure of ~1 GPa^10^. Thus, it is reasonable to speculate that at high Li deposition rate, the interface stress can be rapidly accumulated up to GPa level, in spite of the yielding and creeping of Li. Especially, a crucial question remains unclear: how does a (nearly) defect-free SE respond to this extremely high pressure upon Li plating? All these issues are fundamental for dendrite suppression but yet to be clarified by convincing experiments.

On the other side of the story, the pressure acting on the Li|SE interfaces can play a positive role in achieving a higher CCD. For instance, Li yield under the pressure from a current collector (CC) was presumed to cause horizontal plastic flow to increase the Li|SE interface area of anode-free SSBs, thus promoting the uniformity of Li deposition^19^. Recent works also suggested the positive effect of stack pressure, which can aid in replenishing the Li at the interface and mitigating void formation during stripping^20–23^. However, these hypotheses remain unverified, due to the lack of direct real-time observation of the buried Li|SE interfaces upon charge/discharge.

Herein, we adopt single-crystal LLZO as a model SE to study the above issues by constructing a Li|LLZO|CC nanobattery that allows for cross-sectional observation of the interface dynamics by transmission electron microscopy (TEM). Various interface behaviors are in situ visualized and linked with the Li-deposition-induced stress that relies highly on the local current density and mechanical constraint. We uncover the dual roles of such stresses in stabilizing the Li|LLZO interface: enabling homogeneous Li deposition by creeping (positive role) and initiating the crack and Li penetration in single-crystal LLZO (negative role). The latter offers strong evidence of stress up to GPa or even 10 GPa level, as also confirmed by chemo-mechanical simulations, which can crack LLZO by cleavage regardless of the surface defect size. By weakening the mechanical constraint, we demonstrate the damage-free Li deposition at a current density up to A·cm^−2^ level. Based on these controlled experiments, potential ways to improve the CCD of SEs via fast mass/stress release at the Li|SE interface are also suggested.

## Results

### Low-rate deposition of Li at the LLZO|CC interfaces

**Fig. 1:**
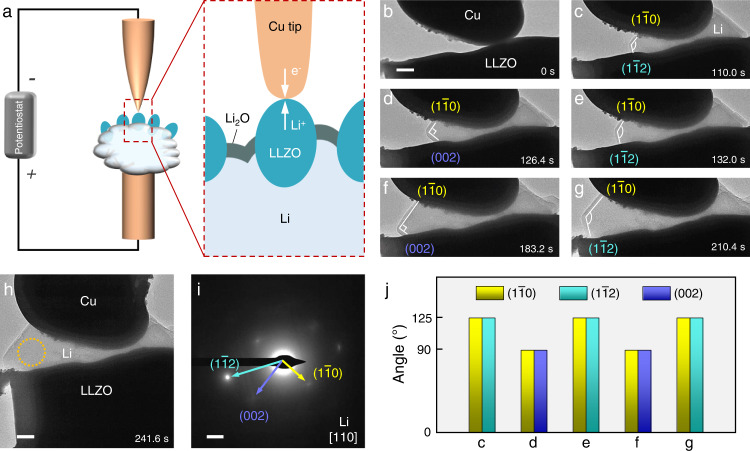
Morphological evolution of single-crystal Li metal growing at the Cu|LLZO interface. **a** Schematic of the anode-free Cu|LLZO|Li nanobattery setup for in situ TEM probing. **b–h** Time-lapsed TEM images revealing the Li growth on the LLZO surface by creeping under a rigid constraint from the Cu current collector at low deposition rate, which enlarged the Li|LLZO interface area. **i** SAED pattern of the plated single-crystal b.c.c. Li. **j** Evolution of crystallographic planes on the growing Li surface and the angle between them in **c–g**. Scale bars: **b**, **h**, 200 nm; **i**, 2 nm^−1^.

The Li|LLZO|CC nanobattery setup in TEM is schematically illustrated in Fig. 1a. A piece of Li metal adhered to a copper rod served as the Li source, and the LLZO particles semi-submerged in Li metal acted as the solid electrolyte. A metal probe was manipulated to form a contact with LLZO to mimic the “hot spots” at the CC|SE interface of anode-free SSBs. Ta-doped LLZO (Li_6.4_La_3_Zr_1.4_Ta_0.6_O_12_) was selected as a model SE for this study. As shown in Supplementary Figs. 1–5, the single-crystal nature of these LLZO particles was revealed by thorough structural, compositional, and size analyses via high-resolution TEM, selected area electron diffraction (SAED), energy dispersive spectroscopy elemental mapping, and scanning electron microscopy. Upon applying a positive potential to the Li source, Li deposition at the CC|LLZO contact can be triggered and visualized by TEM. In a practical SSB, the CC|SE interface is usually uneven, resulting in local contacts and gaps randomly distributed along the interface^19,24^. These contact points tend to become the “hot spots” for Li deposition and even penetration, which normally happens under a certain amount of local pressure. To create such a hot spot at the CC|LLZO interface, different CCs were adopted to create the CC|LLZO interfaces with strong, variable, and weak mechanical constraints (or pressure), respectively (the quantitative evaluations of mechanical constraints of these CCs by in situ mechanical tests are presented in Supplementary Fig. 6).

Figure 1b–h shows a CC|LLZO interface with a strong/rigid constraint by using a thick copper (Cu) probe as the CC. By applying a low constant bias of 0.2 V, Li metal nucleated at the Cu|LLZO contact point, and the deposited Li pushed the Cu probe slightly away from the LLZO, which in turn generated a strong uniaxial pressure. As a result, Li started to grow laterally in between the Cu|LLZO gap (Supplementary Movie 1). The SAED reveals that the plated Li was a single crystal in spite of its irregular morphology under extrusion (Fig. 1i). Such confined growth can be attributed to creep motion of Li metal. Since no clue of dislocations was found, Li^0^ atoms diffusion is believed to account for the lateral extension of Li^16,25^. The adjacent gap space was then gradually filled with Li, leading to increased Li|LLZO interface area. A close examination (based on the SAED analysis) of the Li crystal shows that during its leftward extension, the Li {110} plane was always exposed on the upper growing facet, while {112} and {002} planes emerged alternately on the lower growing facet (both parallel to E-beam, Fig. 1c–h and j). As {110} planes of b.c.c. metals have the lowest surface energy, they are therefore preferentially exposed on the surface of Li crystals, consistent with previous TEM observations^26,27^. Besides, the low-energy Li {002} and {112} planes prevailed alternately on the lower growing facet to adapt to the uneven LLZO surface, so as to minimize the overall energy of the expanding-Li|LLZO system.

**Fig. 2:**
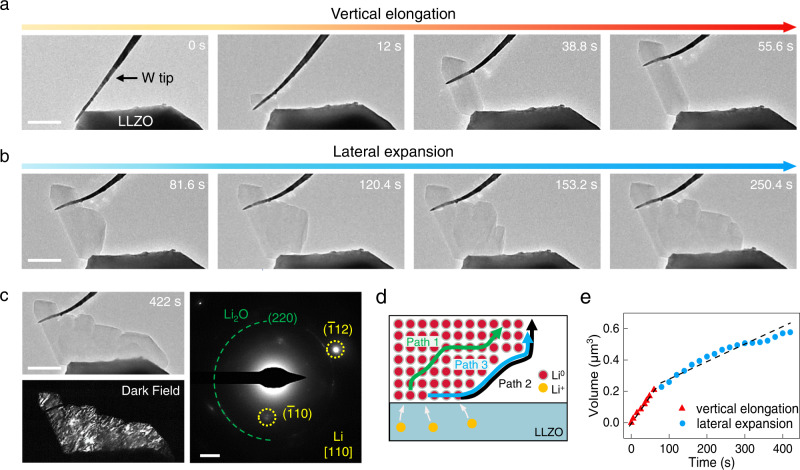
Vertical growth and lateral extension of Li metal under an increasing stack pressure imposed by a slender W current collector. **a** Vertical elongation of a Li whisker by root growth leading to increase in stack pressure from the bending W tip. **b** Lateral swelling (81.6 s) of the Li whisker under a strong compressive stress and the following lateral expansion of Li, which enlarges the Li|LLZO interface. **c** Bright field and dark-field (DF) images of the deposited Li at the final stage of lateral expansion; the DF image is obtained by choosing the diffraction spot of Li $\left(\bar{1}12\right)$ in the SAED pattern, indicating the single-crystal nature of the deposited Li. **d** Schematic of three suggested pathways for Li^0^ atoms diffusion during lateral growth in **b**. **e** Change in volume of the Li metal during plating. Scale bars: **a**, **b** 500 nm; **c** bright image, 500 nm; SAED image, 2 nm^−1^.

In the real situation of SSBs, the CC should be somewhat adjustable in position to accommodate the increasing amounts of Li during plating^28,29^. Therefore, we chose a slender tungsten (W) tip as a flexible CC that can offers a variable constraint (Fig. 2a). Driven by a low bias (0.2 V), Li^+^ ions from LLZO were reduced at the W|LLZO contact, forming a faceted Li particle. As more neutralized Li^0^ atoms were added to the Li|LLZO interface, the Li particle grew into a whisker and push up the W tip, demonstrating a root growth mode^16,25,30^. Note that the Li|LLZO contact area remained almost unchanged (Fig. 2a, from 12 s to 55.6 s), which allows for an estimation of the local current density during the whisker growth to be ~17 mA cm^−2^. The Li whisker continued to elongate until the root growth was terminated by the increasing compression from the W tip, which can reach over 10 MPa (Supplementary Fig. 6) or even higher^16,25^. Thereafter, the whisker began to laterally swell at its shank (Fig. 2b, 81.6 s). Since Li^+^ neutralization and insertion at the Li|LLZO interface generates high compressive stress therein, the accumulated Li^0^ atoms have to transport toward the side surface of the whisker, through bulk or surface diffusion, to release the stress^25^. Figure 2d illustrates three possible Li^0^ diffusion paths. At room temperature, the diffusivity of Li^0^ atoms in bulk (Path 1) is four orders of magnitude lower than that on the free surface (Path 2) (10^−15^ m^2^ s^−1^ vs. 10^−11^ m^2^ s^−1^)^31^. Therefore, the surface diffusion should dominate the lateral deformation of Li. In addition, the generated Li^0^ atoms can first diffuse along the Li|LLZO interface toward the triple-phase boundary (where the Li metal, LLZO, and vacuum meet), followed by faster transport on Li surface, as suggested by path 3.

It is interesting to note that during the shank swelling (Fig. 2b, 81.6 s), Li did not plate at the triple-phase boundary to enlarge the Li|LLZO interface. This indicates that the formation of Li|LLZO interface is not as energetically favorable as expected, since it depends on many factors such as the crystallographic and chemical characteristics of the LLZO surface, as well as the crystal orientation of Li, etc (more discussion of this issue can be found in the Supplementary Discussion). Not until the Li whisker swelled strikingly forming a highly expanded surface, did the Li start to extend laterally to cover the LLZO surface, thus reducing the Li surface area and the system energy (Fig. 2b and Supplementary Movie 2). As revealed by the SAED and dark-field (DF) imaging (Fig. 2c), the deposited Li remained single-crystalline throughout the growth. The comparison of the growth rates shows that the Li vertical elongation was about three times faster than its lateral expansion (Fig. 2e). Obviously, the mechanical constraint plays a vital role in determining the growth pattern and rate. The Li vertical growth started from a relatively weak mechanical constraint and Li^+^ ions are easier to be reduced at the interface. Instead, the entire Li lateral expansion proceeded under large pressure exerted by the highly bent W tip, which should slow down the Li^+^ reduction rate under the same external bias. Intriguingly, despite the small contact area between the W tip and Li metal, the strong compressive stress can be transmitted through the single-crystal Li and act on the whole Li|LLZO interface, thus maintaining the following lateral growth of Li and its intimate contact with LLZO. Moreover, different from the deformation of bulk Li where the dislocation-mediated creep/flow dominates^32^, herein, our results suggest that the diffusion creep is more likely to occur at the local regions of Li|SE interface, where dislocations are difficult to operate within the small-volume Li, as confirmed by recent experiments through nanoindentation^33,34^. Such in situ observations actually offer a rare opportunity to elucidate how the voids at the Li|SE interface are diminished via the lateral extension of the adjacent Li, which helps to alleviate the problem of insufficient contact.

To investigate the Li deposition behaviors under weak or no constraint condition, a carbon nanotube (CNT) was selected as the CC, which was too soft to exert any appreciable constraint on the growing Li. Briefly, Li metal can expand into a faceted particle at a low current density (0.02–0.2 mA cm^−2^) or elongate into a whisker at a relatively high current density (4 mA cm^−2^), exhibiting a rate-dependent growth morphology (see Supplementary Figs. 7 and 8, Supplementary Movies 8 and 9)^35^. Note that without mechanical constraint, the Li crystals showed no conformal growth on LLZO surface, demonstrating again that Li metal is inert toward LLZO and a sufficient pressure is indispensable for enlarging the Li|LLZO interface.

### LLZO cracking and short circuit induced by Li eruption

The Li-deposition induced stress at the Li|SE interface (under a certain amount of compression) can not only profoundly alter the Li growth pattern, but also lead to the failure of SEs. It is well known that SE cracking and Li penetration tend to occur as the current density increases. However, the real causes of this rate-dependent mechanical failure remain unclear. Therefore, the failure of single-crystal LLZO under intense charging was further explored by directly measuring the local current density.

**Fig. 3:**
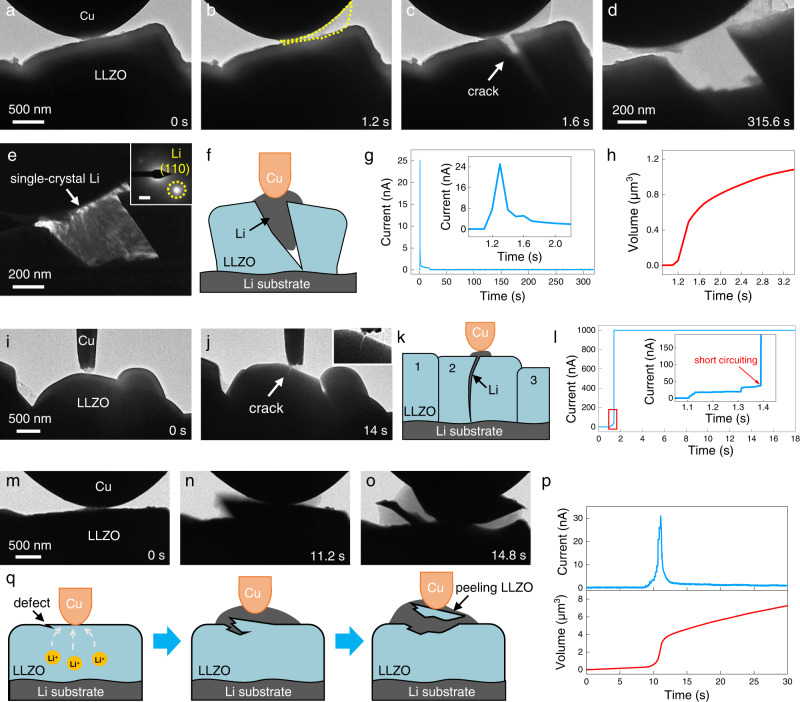
Crack initiation and Li penetration in LLZO by Li eruption at the constrained Cu|LLZO interface. **a–c** Li eruption at the interface to crack a single LLZO particle. **d** The crack was further opened and filled by Li. **e** DF and the corresponding SAED (inset) of the filled Li. **f** Schematic illustration of crack opening and Li filling in LLZO. **g** The measured *I-t* curve. The inset is the enlarged profile of the current peak. **h** The deposition volume change upon Li eruption. **i–j** Li eruption to pierce and short-circuit a LLZO particle in tight contact with the neighboring ones. **k** Schematic illustration of LLZO splitting and Li penetration. **l** The measured *I-t* current curve and the enlarged profile at the sharp current jump that indicates a short circuit. **m–o** Surface peeling of LLZO upon Li eruption. **p** The recorded *I-t* curve and the change of Li deposition volume. **q** Schematic of LLZO surface peeling by the lateral crack propagation in the subsurface of LLZO, leaving behind a dent on the surface.

As shown in Fig. 3a, a thick Cu probe was brought into contact with an isolated LLZO particle, and a high bias of 3 V was then applied to drive Li to rapidly deposit at the Cu|LLZO contact (highlighted by a yellow dashed line in Fig. 3b). Under the strong mechanical constraint imposed by the Cu probe, the fast Li accumulation can generate a huge stress at the contact region. Consequently, a crack filled with Li metal appeared abruptly on the LLZO surface (Fig. 3c and Supplementary Movie 3), accompanied with a prominent peak in the recorded *I-t* curve (Fig. 3g). Upon further Li intrusion, the crack was widely opened (Fig. 3d), leading to the transgranular fracture of the whole LLZO particle, as if splitting a rock in half (Fig. 3f). The DF image and corresponding SAED pattern revealed that the Li metal in the crack was a single crystal (Fig. 3e). The peak current of 25 nA, divided by the measured contact area, can give a rough estimation of the instant current density upon cracking as high as ~3 A cm^−2^, which is at least 3 orders of magnitude higher than the CCD of LLZO. The corresponding Li flux can reach 2.20 μm^3^ s^−1^, as evident from the sharp increase in the overall Li deposition volume at 1.2–1.6 s (Fig. 3h). Such extremely uneven current distribution and fast local Li eruption in a real SSB, if not accommodated appropriately, will unavoidably lead to rapid local stress build-up and SE failure.

Notably, Li intrusion in the split particle can fully penetrate the particle and short circuit the nanobattery. Supplementary Fig. 9 shows an isolated LLZO particle that was gradually opened by the filled Li after splitting, and the full Li penetration, from crack initiation to short circuiting, took a relatively long time of over 3 s. Intriguingly, as for the particle in tight contact with others, the formed crack was constrained from opening and thus the splitting seemed to happen more “quietly” (Fig. 3i, j and Supplementary Movie 4), resulting in a narrow gap that was sometimes “hidden” in the TEM image (if the crack planes are not parallel to e-beam). Careful examination of the enlarged *I-t* curve revealed a stepwise gradual increase in current up to 37 nA before short circuiting, corresponding to the ionic current upon Li eruption and plating (Fig. 3l). The short circuit was evidenced by the sharp jump in current (to its range limit), resulting from the full Li penetration and electrical conduction, which happened in <0.3 s since crack initiation (Fig. 3k). In fact, Li filling along the narrow gap can even lead to superfast Li penetration within 5 ms, corresponding to a penetration speed of at least 0.4 mm s^−1^ (Supplementary Fig. 10 and Supplementary Movie 10).

Besides particle splitting, LLZO can also fracture in the form of surface “peeling”. As demonstrated in Fig. 3m–o and Supplementary Movie 5, two LLZO fragments were peeled off the particle surface induced by high-rate Li plating. Figure 3p shows the corresponding current spike of 31 nA and the deposition volume change of Li. Such kind of fracture may happen as the result of lateral propagation of a crack in the LLZO subsurface. As illustrated in Fig. 3q, the pre-existing surface defects or cleavage planes of LLZO that form a small angle with the particle surface would be preferentially initiated due to the weaker pressure from outside than the inner stress (that is why surface peeling was more frequently observed than particle splitting that happens along a direction roughly perpendicular to the particle surface; more discussion can be seen in Supplementary Fig. 11). During the crack extension in the subsurface, the unbalanced stress further forces the crack to turn its advancing direction toward the particle surface. Eventually, the spalled piece is pushed out by the growing Li, leaving behind a dent on the LLZO surface. Such surface peeling is quite repeatable for LLZO at high Li deposition rate (Supplementary Figs. 12 and 13 and Supplementary Movies 11–14). Note that although the surface peeling does not directly lead to short circuiting of SEs, the spalled pieces and surface dents can become the hot spots to initiate the Li penetration during subsequent cycling.

The Griffith theory is commonly used to judge the crack propagation in brittle materials like LLZO^10,16,36,37^. The critical stress *σ*_*c*_ for crack propagation can be calculated using the following equation^10^:

1 $${\sigma }_{c}\ge \frac{{K}_{{IC}}}{Y\sqrt{\pi a}}$$

where *K*_*IC*_ is fracture toughness, measured to be ~0.95 MPa m^1/2^ for Ta-doped LLZO^38^, *a* is the length of crack, and *Y* is the geometric factor, taken as 1.12^10^. In addition, the deposition overpotential, Δ*φ*, is related to the stress, *σ*, by^10,25^:

2 $$\sigma \cong \,\frac{F}{{V}_{m}}\times \Delta \varphi$$

where *F* is the Faraday constant and *V*_*m*_ is the molar volume of Li metal (13 cm^3^ mol^−1^). We assume that the particles without apparent flaws (see Fig. 3) contain a hidden surface defect near the Cu|LLZO contact before plating. If the defect is 10 nm in length, the critical stress, *σ*_*c*_, should reach 4.8 GPa, with the corresponding Δ*φ* of 0.65 V. Even for a larger defect of 100 nm, the *σ*_*c*_ and Δ*φ* need to be 1.5 GPa and 0.20 V, respectively. Such stress values far exceed the yield strength of Li metal^16,39^, indicating that fast Li deposition in a strongly confined space can produce extreme pressure to initiate cracking, despite the dislocation-mediated deformation (dislocations are likely to be generated under such high stress). Meanwhile, the diffusion creep cannot mitigate the fast stress build-up either due to the slow kinetics, though it works well at low deposition rate as shown above.

**Fig. 4:**
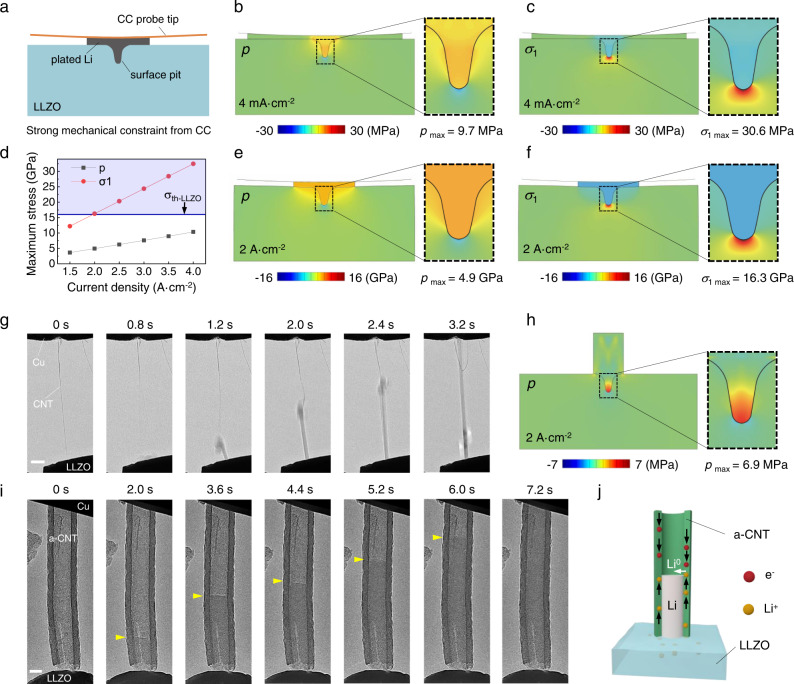
Chemo-mechanical simulated stresses in Li and LLZO and demonstrations of high-rate damage-free Li deposition. **a** Schematic illustration of the chemo-mechanical model under strong top mechanical constraint. The profile of the cross section illustrates that the model consists of a LLZO plate with a surface pit, an arc-shaped current collector (CC) probe tip (orange), and a thin layer of the plated Li between them (gray). **b–c** Distributions of the hydrostatic pressure (*p*) in Li and the maximum principal stress (${\sigma }_{1}$) in LLZO at a low deposition rate of 4 mA cm^−2^. **d** The maximum *p* and ${\sigma }_{1}$ values as a function of current density under high-rate deposition. The line marked by the arrow is the theoretical strength value of LLZO. **e–f** Distributions of *p* and ${\sigma }_{1}$ at a high deposition rate of 2 A cm^−2^ (as an example of the high-rate cases). **g** Fast Li whisker growth induced by a CNT current collector. **h** Distributions of *p* with no top constraint corresponding to the fast Li whisker growth. **i** Fast Li plating inside an amorphous CNT (a-CNT) that serves as a Li host between CC and LLZO. The growth front of Li marked by the yellow arrowhead. **j** Schematic of Li ions transport along the a-CNT wall and their reduction at the Li deposition front. Scale bars: **g** 500 nm; **i** 200 nm.

To further evaluate the Li deposition-induced stresses at the interface, chemo-mechanical simulations were conducted. Simplified model of a LLZO plate with a tiny surface pit interfacing with the deposited Li was built, where different deposition rates under strong top constraint were considered. Figure 4a–f show model setup and the simulated cross-sectional distributions of the maximum principal stress (*σ*_1_) in LLZO and hydrostatic pressure ($p=-\left({\sigma }_{1}+{\sigma }_{2}+{\sigma }_{3}\right)/3$, where ${\sigma }_{1}\ge {\sigma }_{2}\ge {\sigma }_{3}$ are the three principal stresses) in Li. More details of the simulations are provided in Supplementary Method and the corresponding stress evolution is presented in Supplementary Movies 17 and 18. For the case without lateral constraint (equivalent to the case of Fig. 1), the strong top constraint caused the slowly deposited Li to expand along the CC|LLZO interface (4 mA cm^−2^), and the simulated *p* in Li and *σ*_1_ in LLZO at the pit tip are 9.7 MPa and 30.6 MPa, respectively (Fig. 4b, c and Supplementary Movie 17). In contrast, the strong lateral constraint, corresponding to the Li eruption where creep/deformation gets no time to work (2 A cm^−2^, equivalent to the cases of Fig. 3), would generate unanticipated high *p* (4.9 GPa) in Li and *σ*_1_ (16.3 GPa) at the pit tip of LLZO (Fig. 4e, f), with the latter going beyond the theoretical strength of perfect LLZO (16.1 GPa, see Supplementary Discussion). Since *σ*_1_ is highly sensitive to the pit size and geometry, we then place emphasis on the hydrostatic pressure *p* in Li that relies mainly on the current density. As show in Fig. 4d, Supplementary Fig. 16, and Supplementary Movie 18, if *i* is increased to 3 and 4 A cm^−2^, *p* gets boosted to 7.6 and 10.4 GPa, respectively. It can be expected that under such enormous pressure, even the atomic-scale surface defects can initiate the LLZO cracking by cleavage. Considering the fact that the LLZO surface was almost free of visible defects at the fracture sites, it is reasonable to believe that upon Li eruption, the generated huge pressure cracked the LLZO through the atomic-scale surface defects, which were ubiquitous in real SEs (but not easy to resolve under our TEM), leading to the inevitable failure of the single-crystal LLZO as experimentally observed.

### Damage-free Li deposition on LLZO at high current density

To strengthen our understanding on the failure of LLZO, especially the role of mechanical constraint, high-rate Li deposition experiments with a weak constraint were further conducted. As typically shown in Fig. 4g, a CNT protruding from the counter Cu rod was used as a soft CC. The applied high voltage of 3 V enabled immediate nucleation of Li at the CNT|LLZO contact and its fast root growth into a long whisker. It took only 2 s for the Li whisker to elongate by 3.8 μm until touching the Cu rod (Fig. 4g, from 1.2 s to 3.2 s, see Supplementary Movie 6). The average growth rate of the Li whisker was measured to be 1940 nm s^−1^, corresponding to a current density of ~1.44 A cm^−2^ (the peak current density reached ~4 A cm^−2^). Remarkably, despite the high current density that is comparable to the above cases in Fig. 3, no crack initiation on the LLZO surface was observed. This clearly demonstrates that high local current density, even up to A cm^−2^ level, does not necessarily lead to a damage to the SE surface. Such damage-free Li deposition is highly repeatable, which is also solid evidence showing that the high pressure (under a strong constraint), instead of SE defects or local current density, is the primary reason for SE damage (we did not even observe a single event of SE damage that happens under high current density but a weak constraint).

The above controlled experiments highlight the critical role of the applied mechanical constraints that can significantly change the growth mode of Li and, accordingly, the way of mass/stress release at the interface. Instead of the sluggish lateral creep under strong uniaxial constraints, here, the deposited Li leaves the interface instantly with the vertical growing whisker, which can effectively avoid the stress build-up. We then consider the growth stress *σ* at the interface and the associated overpotential Δ*φ* that drives such fast whisker growth, which can be linked with the growth velocity of the whisker *v* by the equation as we propose below (see Supplementary Discussion):

3 $$\frac{F\triangle \varphi }{{V}_{m}}{dV}=\frac{\gamma s}{A}{dV}+\frac{1}{2}{\rho }_{{Li}}{v}^{2}{dV}$$

where *γ* is the surface energy of the Li whisker in vacuum, simply taken as 0.452 J m^−2^ ^40^, *s* and *A* are the perimeter and area of the contact, *ρ*_*Li*_ is the density of Li, and *dV* represents the deposition volume. By substituting the measured *v* into Eq. 3, we obtain the overpotential of 1.65 mV and the corresponding stress of ~12 MPa. Furthermore, chemo-mechanical simulations also reveal that fast vertical growth of Li with no constraint produces limited stress at the interface of *p* = 6.9 MPa (Fig. 4h, Supplementary Fig. 17, and Supplementary Movie 19). Such a low stress confirms that the free growth of Li can hardly cause any damage to the LLZO particles, consistent with our observations.

Both the experimental results and theoretical analysis indicate that a crack would be inevitably initiated at the Li|SE interface, almost irrespective of the SE structural quality, due to the superfast mass/stress accumulation within the constrained space. This suggests that fast Li transport/diffusion away from the hot spot is the key for achieving quick stress release and improved rate capability of SEs (more evidences are provided in Supplementary Figs. 14 and 15, Supplementary Movies 15 and 16). In the light of this principle, some potential ways are herein proposed to address the issue. One method is the use of Li hosts, which can accommodate the volume changes of Li metal and allow efficient Li^+^ transport out of the host|SE interface. We present a simple demonstration in Fig. 4i, j, where the amorphous carbon nanotube (a-CNT) serves as a Li host between LLZO and CC^27^. Li metal can quickly fill the cavity of a-CNT through Li^+^ (or Li^0^ atoms^31^) diffusion along the carbon shells with an estimated current density of 200 mA cm^−2^ (Supplementary Movie 7), still much faster than the cases in Figs. 1 and 2. This means that Li transport by Li^+^ diffusion along a carbon-based host is more efficient than Li creep at room temperature. Another way is to increase the working temperature, which can promote Li^0^ diffusivity as well as the transition in viscosity. In fact, the feasibility of this strategy has been verified by recent works using molten Li anode^41^. A high CCD of 530 mA cm^−2^ was reported for molten Li|LLZO, which was attributed to the pressure relaxation associated with the aforementioned changes in the mechanical properties of Li at elevated temperature (as also confirmed by the chemo-mechanical simulations in Supplementary Fig. 18 and Supplementary Movie 20).

Finally, we need to point out that our in situ tests based on the nanobattery setup are not intended to represent the whole picture of the Li|SE interfaces at bulk length scale, but to reveal some features typical of local Li|SE contacts. Despite some extreme circumstances we created in situ that might not be common in real SSBs, we believe that such in situ tests can still provide valuable information for understanding the Li nucleation/growth and dendrite initiation in SEs from a scientific standpoint.

## Discussion

To conclude, by creating a cross-sectional view of Li|LLZO interface in TEM, we revealed the interface dynamics and associated SE failure mechanism. Various interface behaviors can be attributed to the Li-deposition induced stress that relies highly on the charging rate and mechanical constraint. We provided the direct observation of creeping deformation of single-crystal Li, which enlarges the Li|LLZO interface at high stack pressure and low deposition rate. Impressively, the crack of LLZO under rigid constraints, in the form of “splitting” or “peeling”, is clearly demonstrated and linked with a prominent current pulse passing through these hot spots. These results, combined with chemo-mechanical simulations, reveal that extreme stress of 10 GPa level is probably generated, which can even crack a nearly defect-free LLZO particle by cleavage. Inspired by our findings, possible strategies that can boost the local current density without damaging the LLZO via fast mass/stress release from the Li|LLZO interface are also suggested, such as quick Li^+^ transport through carbon hosts and promoted Li^0^ diffusion at high temperature. This work offers not only valuable insights for understanding the rate-dependent stresses and the resultant SE failures, but also a stress-release-oriented guideline that enables fast charging of SSBs.

## Methods

### Synthesis of Li_6.4_La_3_Zr_1.4_Ta_0.6_O_12_ (LLZO)

The cubic garnet-type solid electrolyte Li_6.4_La_3_Zr_1.4_Ta_0.6_O_12_ (LLZO) was synthesized with a solid phase method. Briefly, LiOH·H_2_O (95%, Sinopharm), La_2_O_3_ (99.99%, Sinopharm), ZrO_2_ (99.99%, Aladdin), and Ta_2_O_5_ (99.8%, Aladdin) were mixed in stoichiometric ratio with 15 wt% excess of LiOH·H_2_O, and the mixture was then calcined at 900 °C for 12 h in alumina crucible to obtain pure phase material. The as-prepared particles were sintered into ceramic pellet to measure the ionic conductivity, and the highest conductivity is 6.9 × 10^−4 ^S cm^−1^, as reported in our recent work^6^.

### In situ TEM characterization

The in situ TEM experiment was carried out inside a FEI Talos-F20s TEM at an accelerating voltage of 200 kV. The beam dose was 0.16–2.5 e Å^−2^ s^−1^ (i.e., 0.26–4 mA cm^−2^) for in situ imaging, which did not bring any obvious damage to the samples. The TEM was equipped with a TEM-STM holder (ZepTools Co. Ltd., China), which was capable of piezo-driven manipulation and electrical biasing. In a glove box filled with Ar gas, LLZO particles were first distributed on a glass substrate. Then a Cu rod mounted with Li metal was pressed onto the substrate, and the particles can be attached and submerged in the Li metal, which was then mounted on one end of the holder. A metal probe, which can be a Cu tip, sender W tip or Cu tip attached with CNTs, was mounted onto the other end of the holder to serve as a working electrode, as well as provide different mechanical constraints for in situ electrochemo-mechanical tests. The holder was then inserted into the TEM, during which a thin oxidation layer was formed on the Li metal surface due to a few seconds of exposure to air. After the working electrode was brought into contact with a LLZO particle, an anode-free solid-state nanobattery was in situ assembled, enabling a cross-sectional view of the interfaces. Then, a constant bias was applied to the Li metal counter electrode to initiate the Li deposition process.

### Chemo-mechanical simulations

Chemo-mechanical simulations were conducted to explore the effects of Li deposition rate and mechanical constraint on the stress generation and subsequent failure of SEs. Figure 4a shows the cross section of our simulation system, which consists of a LLZO plate with a surface pit, an arc-shaped CC probe tip, and a thin “interphase layer” between the LLZO and CC that represented the plated Li. During simulation, the CC probe tip was assumed to be a rigid surface that can exert strong mechanical constraint on Li. The LLZO and the plated Li were treated as an isotropic linear elastic and elastic-viscoplastic materials, respectively. All the materials properties were specified according to experimental data whenever possible. For the plating of Li, instead of explicitly simulating the migration process of Li, the Li deposition process was analogous to the swelling of the thin interphase layer with the fiber-growth mode. With pertinent boundary and initial conditions, the Li plating problem was finally solved by the temperature-displacement procedure in ABAQUS/Standard.

## Supplementary information

Supplementary Information

Description of Additional Supplementary Files

Supplementary Movie 1

Supplementary Movie 2

Supplementary Movie 3

Supplementary Movie 4

Supplementary Movie 5

Supplementary Movie 6

Supplementary Movie 7

Supplementary Movie 8

Supplementary Movie 9

Supplementary Movie 10

Supplementary Movie 11

Supplementary Movie 12

Supplementary Movie 13

Supplementary Movie 14

Supplementary Movie 15

Supplementary Movie 16

Supplementary Movie 17

Supplementary Movie 18

Supplementary Movie 19

Supplementary Movie 20

## Data Availability

The authors declare that all the relevant data are available within the paper and its Supplementary Information file or from the corresponding author upon reasonable request.
