# Supplementary material for: Visualizing the failure of solid electrolyte under GPa-level interface stress induced by lithium eruption
Source: Nat Commun. 2022 Aug 27;13:5050. doi: 10.1038/s41467-022-32732-z (PMC9420139; doi:10.1038/s41467-022-32732-z)
Supplement: Supplementary file 1 — Supplementary Information [file 41467_2022_32732_MOESM1_ESM.pdf]

Supplementary Information

Visualizing the Failure of Solid Electrolyte under GPa-level Interface Stress Induced by Lithium Eruption

Haowen Gao[1], Xin Ai[2], Hongchun Wang[3], Wangqin Li[1], Ping Wei[1], Yong Cheng[1], Siwei Gui[2], Hui Yang[2,*], Yong Yang[3], Ming-Sheng Wang[1,*]

[1]State Key Laboratory of Physical Chemistry of Solid Surfaces, College of Materials, Xiamen University, Xiamen, 361005, China

[2]Department of Mechanics, School of Aerospace Engineering, Huazhong University of Science and Technology, Wuhan, Hubei 430074, China

[3]State Key Laboratory of Physical Chemistry of Solid Surfaces, College of Chemistry and Chemical Engineering, College of Energy, Xiamen University, Xiamen, Fujian 361005, China

** Corresponding Author,*

E-mail: huiyang2017@hust.edu.cn (H.Y.), mswang@xmu.edu.cn (M.-S. W)

Supplementary Information includes:

1. **Supplementary Method**
2. **Supplementary Discussions**
3. **Materials characterizations: Supplementary Figs. 1-5**
4. **Supplementary in situ TEM Results: Supplementary Figs. 6-15**
5. **Chemo-mechanical simulations: Supplementary Figs. 16-18**
6. **Supplementary Movie Captions: Supplementary Movies 1-20**

1. Supplementary Method

Detail of Chemo-mechanical simulations

Chemo-mechanical simulations were conducted to explore the effects of Li deposition rate and mechanical constraint on the stress generation and subsequent failure of SEs. Fig. 4a shows the cross section of our simulation system, which consists of a LLZO plate with a surface pit, an arc-shaped current collector (CC) probe tip, and a thin "interphase layer" between the LLZO and CC that represented the plated Li. During simulation, the CC probe tip was assumed to be a rigid surface that can exert strong mechanical constraint on Li. The LLZO was treated as an isotropic linear elastic material with Young's modulus E and Poisson's ratio v equals to 140 GPa and 0.26, respectively.[1] For the plating of Li, instead of explicitly simulating the migration process of Li, the Li deposition process was analogous to the swelling of the thin interphase layer with the fiber-growth mode.[2,3] The elastic-viscoplastic behavior of plated Li was then described by the Anand model,[4] in which the flow equation is given by:

$$\dot{\varepsilon}^p = A\exp\left(-\frac{Q}{RT}\right)\left[\sinh\left(\xi\frac{\tilde{\sigma}}{s}\right)\right]^{\frac{1}{m}} = \dot{\varepsilon}_0\left[\sinh\left(\xi\frac{\tilde{\sigma}}{s}\right)\right]^{\frac{1}{m}} \tag{S1}$$

where $\dot{\varepsilon}^p$ is the inelastic strain rate, A is the pre-exponential factor, m is the strain rate sensitivity, Q is the activation energy, R is the universal gas constant, T is the absolute temperature, $\tilde{\sigma}$ is the equivalent stress, ξ is the material constant, and s is the deformation resistance. With the initial value of s_0, the deformation resistance s evolves as:

$$\dot{s} = h_0\left|1-\frac{s}{s^*}\right|^a sign\left(1-\frac{s}{s^*}\right)\dot{\varepsilon}^p \tag{S2}$$

where h_0 is the hardening/softening constant and a is the strain rate sensitivity of hardening/softening. s^* represents the saturation value of s and can be expressed as:

$$s^* = \hat{s}\left[\frac{1}{A}\dot{\varepsilon}^p \exp\left(\frac{Q}{RT}\right)\right]^n \tag{S3}$$

where $\hat{s}$ is a coefficient, and n is the strain rate sensitivity for the saturation value of s. By fitting available experimental data,[5] the pertinent parameters for the Anand model can be obtained, as listed in the Table S1.

According to the Li plating current density i, the corresponding Li flux can be calculated as $j = i/F$, where $F = 9.6485\times10^4$ C/mol is the Faraday constant. If the contact area of LLZO and interphase Li layer is Δa and the volume of the thin interphase layer is Δv, the balance of the deposited Li gives $j\Delta a = \dot{c}_\mathrm{R}\Delta v$, which can lead to the evolution of Li concentration c_R in the interphase layer as $\dot{c}_\mathrm{R} = \frac{i}{F}\frac{\Delta a}{\Delta v}$. Assuming Li plating can cause a 500% volume change of the interphase layer, the maximum Li concentration can be derived by $c_\mathrm{Rmax} = \frac{5}{\Omega}$, where $\Omega = 13\times10^{-6}\ \mathrm{m^3/mol}$ is the molar volume of Li.[2] Then, the evolution of normalized Li concentration is $\dot{\bar{c}} = \frac{\dot{c}_\mathrm{R}}{c_\mathrm{Rmax}} = \frac{\Omega}{5}\frac{i}{F}\frac{\Delta a}{\Delta v}$. Assuming next the current density i along the local LLZO|Li interface is uniform so as to simplify our simulation, for the given geometry of our model and when i equals 0.004, 1.5, 2.0, 2.5, 3.0, 3.5, and 4.0 A·cm^{-2}, the corresponding $\dot{\bar{c}}$ can be estimated as 0.006, 2.245, 2.994, 3.743, 4.491, 5.240, and 5.998, respectively. When the deposition current density i is small enough, i.e. $i = 0.004$ A·cm^{-2}, the deposited Li has sufficient time to migrate to the nearby region, therefore the lateral surface of the interphase layer was set to be free. However, for the other cases that $i \geq 1.5$ A·cm^{-2}, the lateral surface of the interphase layer was fixed as the plating of Li is too fast in the local region that Li lateral migration is negligible. To investigate the mechanical constraint effect on the stress generation in LLZO, the lateral surface of the interphase layer for another case with $i = 2.0$ A·cm^{-2} was deliberately set to be free for comparison. In addition, the CC probe tip and the bottom of the LLZO were fixed. Due to symmetry of the system, half of the cross section with plane strain

condition was simulated to reduce the computational cost. With the initial condition that $\bar{c} = 0$, the Li plating problem was finally solved by the temperature-displacement procedure in ABAQUS/Standard.

Table S1 Fitted elastic-viscoplastic material parameters for Li.

E (GPa)	7.81
ν	0.38
A (s^{-1})	2.5988×10^4
Q (kJ/mol)	37
m	0.16
ξ	1.25
s_0 (MPa)	1.2703
h_0 (MPa)	8.5638
$\hat{s}$ (MPa)	2.0013
a	1.0649
n	0.07

2. Supplementary Discussions

The discussion on the lithiophilicity of LLZO surface

As discussed in Fig. 2, the formation of Li|LLZO interface is not a spontaneous process, but requires a strong pressure. This can be confirmed by the controlled experiment under weak or no mechanical constraint, where the Li crystals showed no conformal growth on the LLZO surface (Supplementary Figs. 7 and 8). Even under the strong pressure, the Li crystal would first swell at its shank (thus increasing the surface energy), rather than plate at the triple-phase boundary to enlarge the Li|LLZO interface (Fig. 2b), which also indicates that Li metal is actually inert toward LLZO.

This seems to contradict with the conclusion that the LLZO surface is lithiophilic, as proposed in previous experimental studies.[6,7] Here, we need to point out that in the experiments to test the lithiophilicity of LLZO, molten Li was adopted, and its wettability with LLZO was evaluated by measuring their contact angle and linked with the lithiophilicity. However, such measurements cannot reflect the lithiophilicity of the solid-solid interface, which is representative of solid-state cells that are generally cycled at room temperature. Indeed, the low interface resistance and the intimate contact between Li and LLZO need chemical bonding such as the formation of the Li-O bond at the interface.[8] However, there are some prerequisites for LLZO to exhibit such lithiophilicity. For example, the construction of the chemical bond usually requires a relatively high temperature above the Li melting point or a sufficiently high pressure to overcome the energetic barrier.[6,7,9,10]

In addition, as mentioned in our manuscript, the lithiophilicity of the LLZO surface is highly sensitive to many factors, such as the crystallographic and chemical characteristics of the LLZO surface, as well as the crystal orientation of Li, etc, which is also suggested by the theoretical study.[8] Specifically, one may question about the chemical property of the LLZO surface, such as the formation of Li_2CO_3, etc. In our in-situ experiments, we always chose the LLZO particle with clean surface, which is significantly different from those covered by a Li_2CO_3 layer (see Supplementary Figs. 2-3). Nevertheless, we can not fully rule out the possibility of the formation or

adsorption of hetero-species at atomic scale, which could also influence the lithiophilicity of LLZO surface.

To conclude, our in-situ experiments did demonstrate that the Li|LLZO interface was not as energetically favorable as expected. This suggests that the LLZO surface is intrinsically not affinitive to solid Li (different from the results based on liquid-solid contacts at macroscale), or there were some factors that reduced the lithiophilicity of LLZO, as stated above.

Linking the overpotential to the whisker growth velocity

Driven by an overpotential $\Delta\varphi$, Li^+ ions from LLZO are reduced at the Li|LLZO interface and the deposited Li is then lifted away from the interface with a velocity v. During such free growth of Li whisker, the electrical work $nF\Delta\varphi$ (n is the amount of deposited Li in a given moment, and F is the Faraday constant) is converted to the surface energy and the kinetic energy of the newly deposited Li. Here, the amount of the Li could be expressed by $n = dV/V_m$, where dV is the volume of the deposited Li in the moment and V_m is the molar volume of Li. The increase in surface energy is $\gamma s dL$, where γ represents the unit surface energy of the Li whisker in vacuum, s is the perimeter of the contact, and dL is the elongation of the whisker in the moment, which can be replaced by dV/A (A is the contact area). The kinetic energy is $\frac{1}{2}\rho_{Li} dV v^2$, which means that the freshly generated Li with a mass of $\rho_{Li} dV$ is accelerated from rest to a velocity v. Thus, according to the law of conservation of energy, the relationship between the overpotential and the velocity of whisker growth can be described as:

$$\frac{F\Delta\varphi}{V_m} dV = \frac{\gamma s}{A} dV + \frac{1}{2}\rho_{Li} v^2 dV \quad \text{(S4)}$$

In addition, we also consider the influence of gravity during the whisker growth. We assume that the growth direction is opposite to gravity, which should impose the maximum stress on the interface by gravity. In this situation, the work of gravity in a certain moment is $mgdV/A$, where m is the mass of the grown whisker. However, the calculated work of gravity is nine orders of magnitude lower than the increase of surface

energy, so its influence on the overpotential is negligible. As a matter of fact, detailed calculations using Eq. S4 show that the growth velocity also has little influence on the overpotential, as well as the corresponding stress at the interface, indicating that free growth of Li will not bring damage to the SE.

The theoretical strength of perfect cubic LLZO

The theoretical fracture strength of LLZO can be considered as the stress to cleave the perfect single-crystal LLZO. The actual strength of LLZO should be lower than this theoretical value due to the presence of interior or surface defects. Calculation of the theoretical fracture strength is important for evaluation of the maximum stress by Li deposition required for cracking and penetrating solid electrolytes. During the brittle fracture of a single crystal, the low-index planes are prone to be cleaved, due to their large interplanar spacing. As a typical brittle material, the theoretical strength $\sigma_{th\text{-}LLZO}$ of the perfect cubic LLZO can be calculated by:[11,12]

$$\sigma_{th-LLZO} = \left(\frac{E\gamma}{d}\right)^{\frac{1}{2}} \tag{S5}$$

where E is the Young's modulus, taken as 140 GPa for LLZO,[1] γ is the surface energy of the cleavage plane, and d is the interplanar distance. Theoretically, the {220} planes, as one of the low-index planes of LLZO, are most likely to be cleaved. According to previous results, the surface energy of {110} plane, equivalent to {220} planes of LLZO, is calculated to be 0.85 $J \cdot m^{-2}$, and the interplane distance is 0.456 nm.[8] Thus, the theoretical strength of perfect cubic LLZO is estimated to be about **16.1 GPa**. This means that the Li-deposition induced stress above this critical value can crack LLZO by cleavage, regardless of its structural perfectness.

3. Materials characterizations

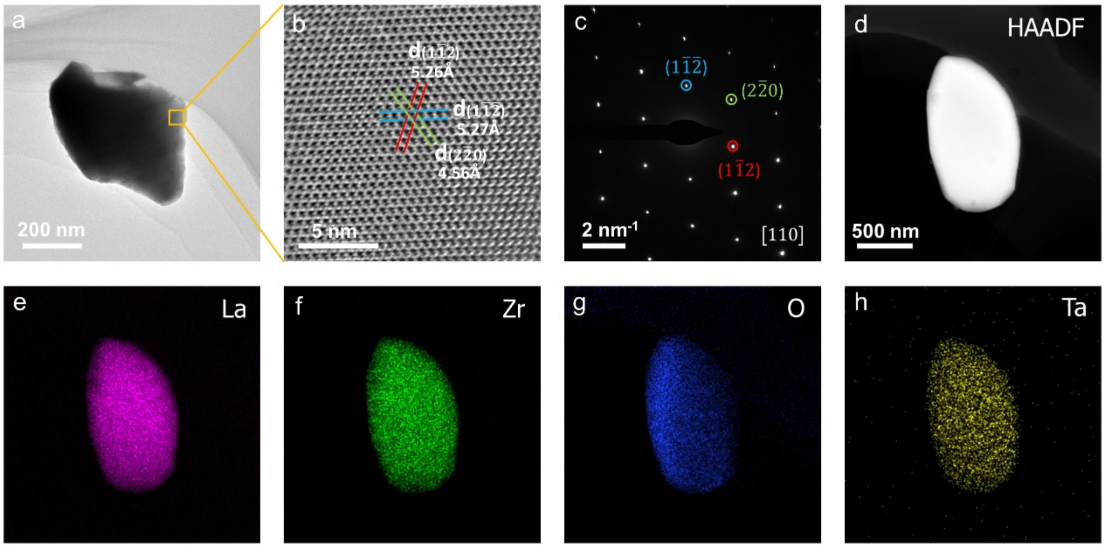

Supplementary Fig. 1 | Morphological and structural characterization of LLZO particles. TEM image of a LLZO particle (a) and the corresponding HRTEM and SAED pattern along [110] zone axis (b-c). (d-h) HAADF image and the corresponding EDS element mapping of another LLZO particle.

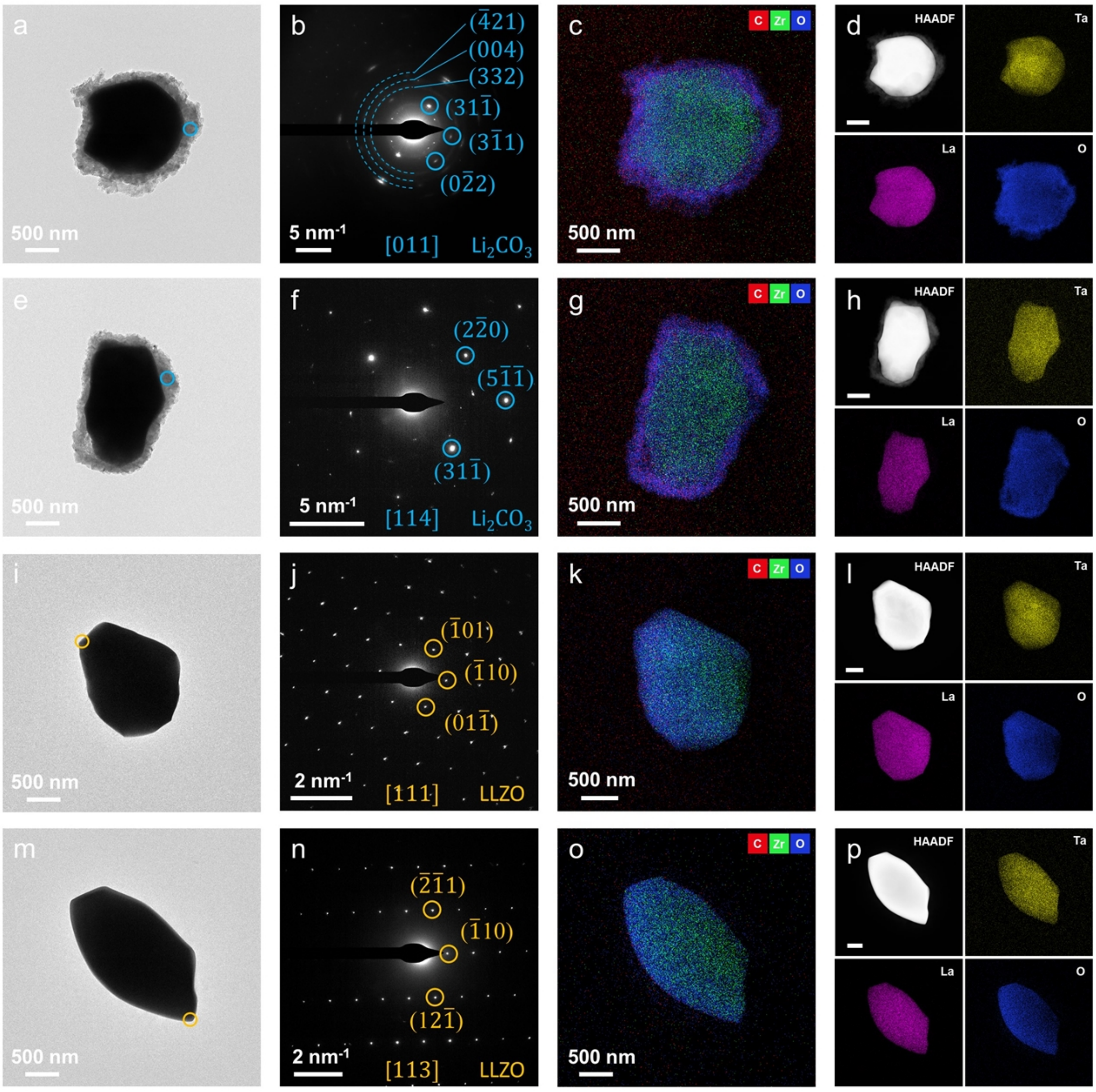

Supplementary Fig. 2 | Structural and elemental characterization of the LLZO particles with contaminated and clean surface by TEM. (a) Morphology of a contaminated LLZO particle after exposure to the air for one week. (b) The SAED pattern acquired from the surface contamination layer in the circled region in (a), which is assigned to Li_2CO_3. (c) Overlap of the EDS maps of C, Zr, O elements for the contaminated LLZO particle indicates a contaminated layer rich in carbonate. (d) The HADDF image and the EDS maps of Ta, La and O of the contaminated LLZO particle. (e-h) The same TEM characterization of another contaminated LLZO particle. (i) The morphology of a clean LLZO particle. (j) The SAED pattern acquired from the edge of the LLZO in (i) indicates a pure phase of single-crystal LLZO, without any impurities. (k) Overlap of the EDS maps of C, Zr, O elements also confirms the clean LLZO surface free of contamination. (l) The HADDF image and the EDS maps of Ta, La and O of the clean LLZO particle. (m-p) The same TEM characterization of another clean LLZO particle. The scale bars in (d), (h), (l), (p) are 500 nm.

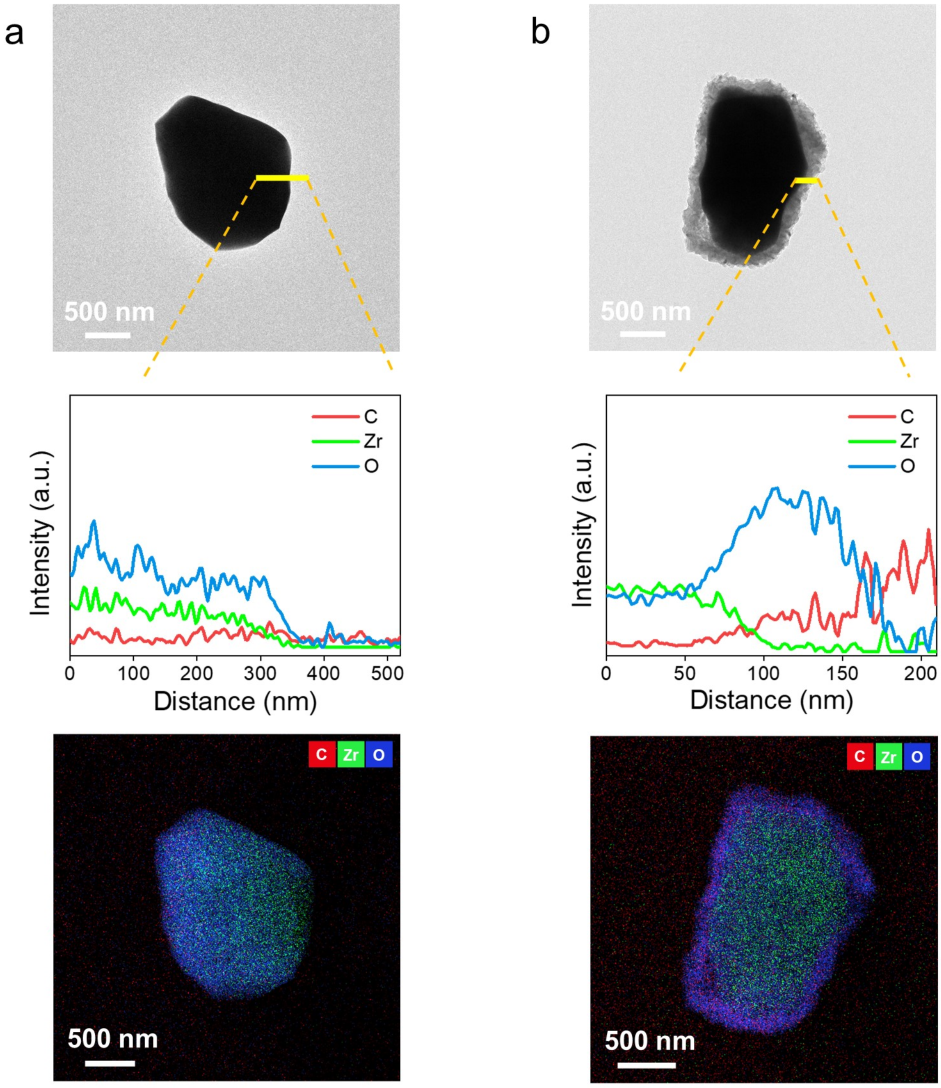

Supplementary Fig. 3 | Elemental analysis of the clean and contaminated LLZO particle by EDS line scan and mapping.

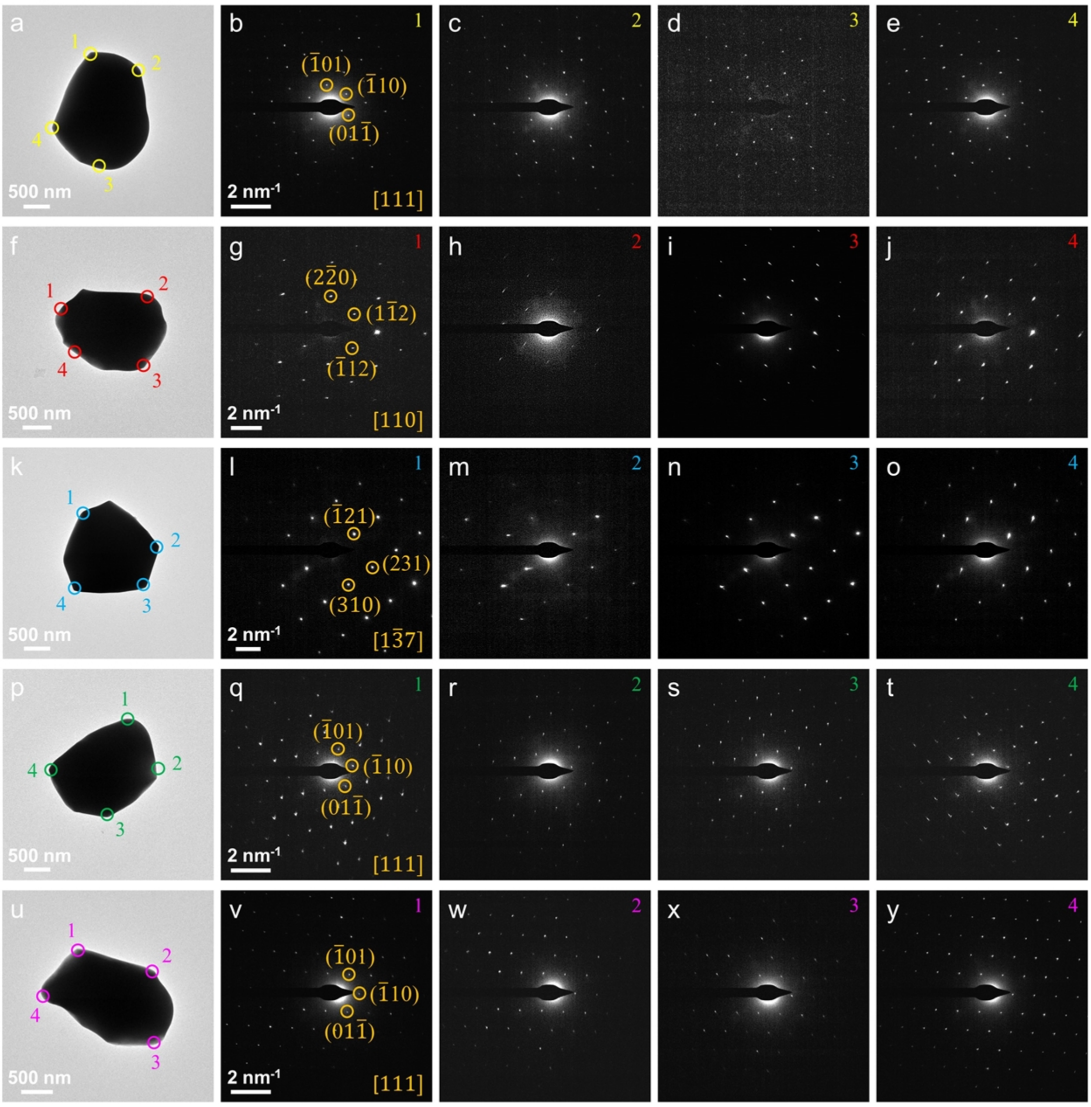

Supplementary Fig. 4 | Single-crystal cubic-phase LLZO particles as confirmed by SAED analysis (a) The morphology of a LLZO particle and (b-e) the corresponding SAED pattern from four edge sites of this particle. The same crystallinity characterization performed on other four particles are shown in (f-j), (k-o), (p-t), (u-y), respectively.

In order to probe the crystallinity of the LLZO particles used for our in situ TEM experiments, we performed a thorough structural characterization by SAED, as shown in Supplementary Fig. 4. These LLZO particles are mostly micrometers in size, which are too thick for the electron beam to pass through. We therefore chose the relatively thin edge regions of each particle for SAED characterization. The SAED patterns acquired from four different edge sites of a particle confirm that all these domains have the same crystal orientation, indicating the single-crystal nature of the particle. All the five LLZO particles examined in Supplementary Fig. 4 are single-crystalline, and we can therefore reasonably believe that the LLZO particles for in situ TEM experiments should be single-crystalline as well.

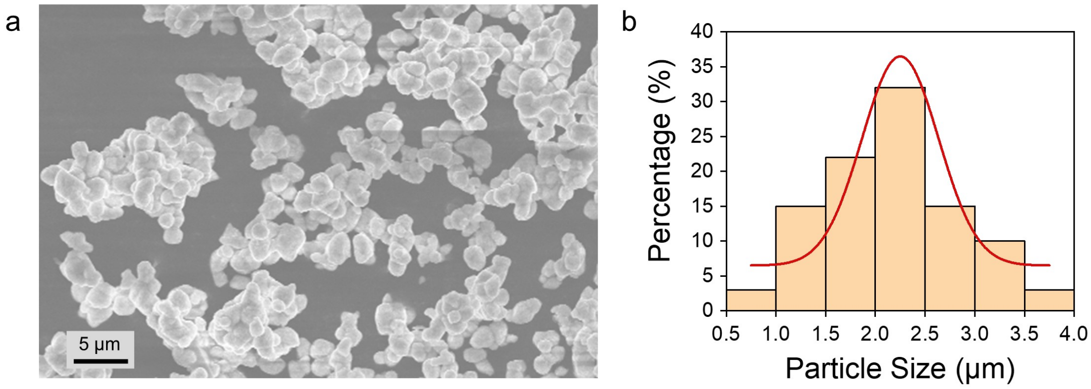

Supplementary Fig. 5 | SEM image of as-synthesized LLZO electrolyte in (a) and corresponding particle size distribution in (b). The distribution of the LLZO particle size, ranging mostly from 0.8 μm to 3.7 μm, and the average particle size is 2.2 μm.

4. Supplementary in situ TEM Results

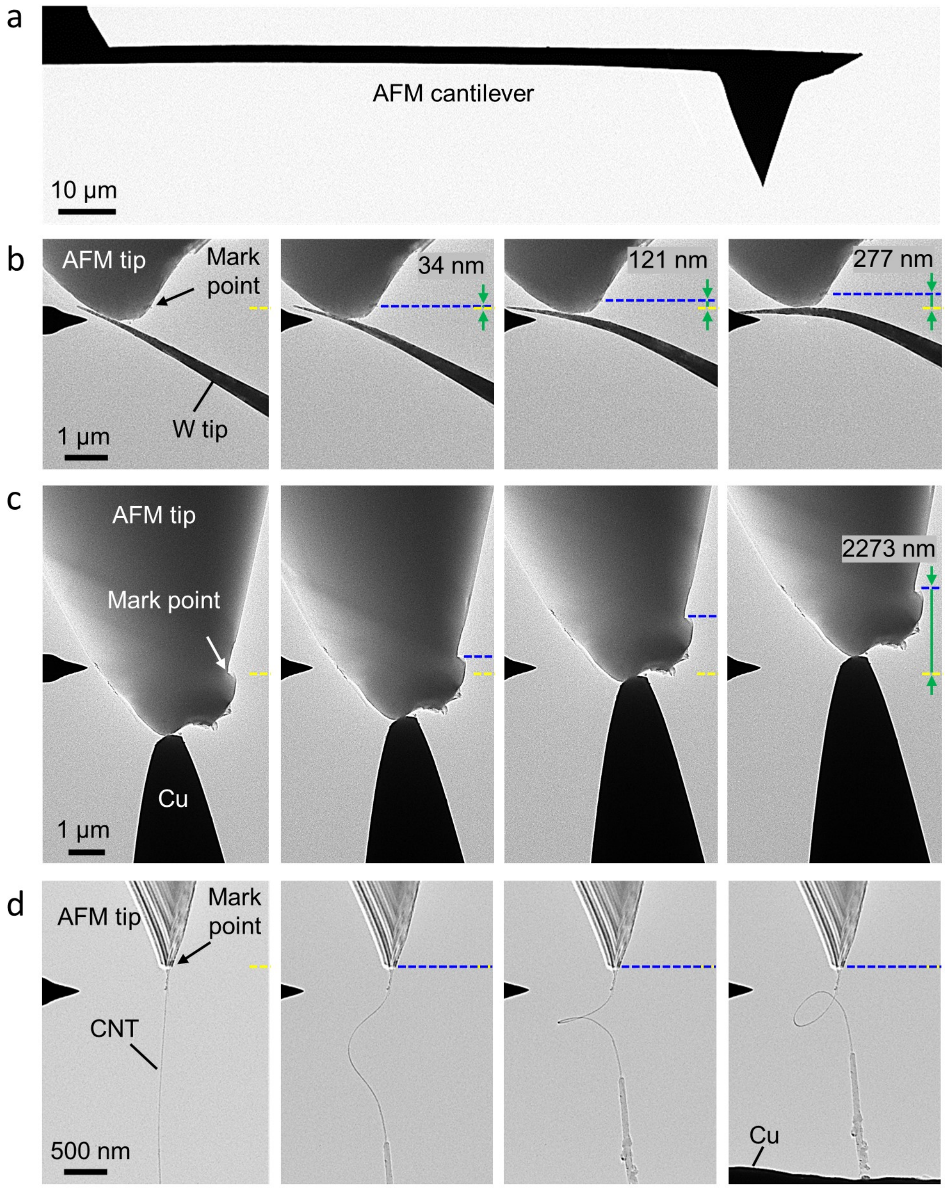

Supplementary Fig. 6 | In situ TEM-AFM compression tests of the three different probes. **a**, Low-mag TEM image of the AFM cantilever. The spring constant of the Si AFM cantilever was k=5 N m^{-1} in all tests. **b**, Compression test of a slender W tip. **c**, The strength measurement of a rigid Cu probe, which shows no obvious deformation under the high compressive force. **d**, Compression test of a slender CNT, which was bent and curved under compression. The two ends of CNT were welded onto the AFM tip and the Cu rod by e-beam-induced carbon deposition, respectively.

To quantitatively measure the stack pressure exerted by the three different probes (Cu, W, CNT), in situ TEM force measurements of these probes were performed by using a TEM-atomic force microscopy (AFM) holder. An AFM cantilever was mounted on the stationary side of the

TEM holder, while a probe tip connected to a piezo-controller was moved to touch the cantilever tip and push it moved upward (Supplementary Fig. 6). The force exerted by the AFM cantilever can be calculated by Hooke's law, $F= k\cdot\Delta x$, where k is the spring constant of the AFM cantilever ($k = 5\ N/m$ in our tests) and Δx is the displacement of the cantilever tip.

As shown in Supplementary Fig. 6b, a slender W tip was forced to bend under the compression force by the cantilever tip. Obviously, the force is not constant and would increase with the displacement of the cantilever tip. As the Δx value increased to 277 nm, the deflection degree of the W tip was close to that in Fig. 2, and the corresponding force was 1385 nN. Here, we can only roughly estimate the force and give the order of magnitude of the stack pressure mentioned in the main text. For the experiment in Fig. 2, if we assume the vertical growth of the whisker was terminated at the maximum force, i.e. around 1385 nN, then the maximum stack pressure exerted by the W tip was ~13 MPa, which in the range of previously reports.[13,14]

In contrast, the Cu probe exhibited no deformation during the in situ test (Supplementary Fig. 6c), though it pushed up the AFM tip with a large displacement of 2273 nm, corresponding to a huge compression force of 11365 nN (it can be even larger if the Cu probe continues to move forward), which is about one order of magnitude higher than the slender W tip. This experiment demonstrated clearly that the thick Cu probe we used was rather rigid and can impose a high pressure at the LLZO|Li|Cu interface, which, of course, also depends on the Li deposition rate.

As for the case of CNT, we also conducted the in situ mechanical test as shown in Supplementary Fig. 6d, where the compression of the nanotube did not cause any visible displacement (less than 1 nm) of the cantilever tip before the buckling of the nanotube. This indicates that the stack pressure exerted by a CNT is indeed negligible. Moreover, we can also estimate the maximum force in the CNT, that is, the axial force that causes the buckling of nanotube in i.e. Fig. 4g by the Euler buckling force formula, $F_{Euler} = \pi^2 EI/L^2$, where E is the Young's modulus, I is the moment of inertia ($I = \pi (d_2^4 - d_1^4)/64$), and L is the length of the CNT between the two contacts.[15] Even E was assumed to be as large as 1 TPa (usually less than 1 TPa), F_{Euler} is estimated to be 0.23 nN. This value is at least 3-4 orders of magnitude lower than the W tip and Cu probe, and that is why no appreciable deflection of the cantilever was observed**.** Thus, we can reasonably believe that a slender CNT can impose almost no pressure on the growing Li crystals, as stated in our manuscript.

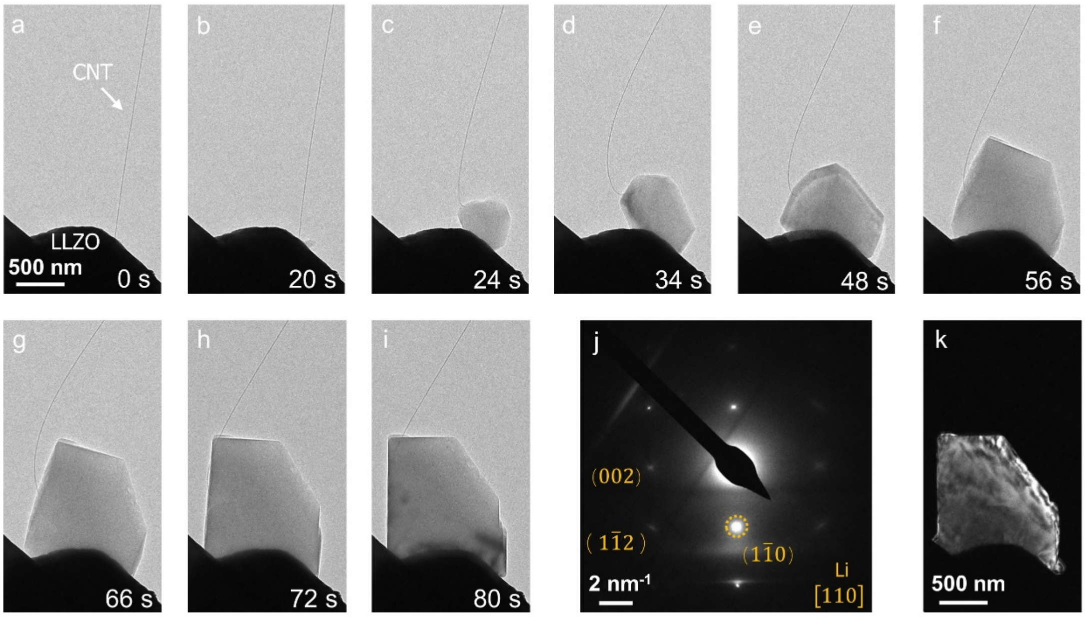

Supplementary Fig. 7 | Nucleation and free growth of a Li particle on the LLZO surface with a CNT current collector (a-i) Time-lapse TEM images of a Li deposit that nucleated at the CNT|LLZO contact point and gradually grew into a faceted particle. (j) SAED pattern revealing the single-crystal structure of the Li particle. (k) Dark-field (DF) image formed by choosing the diffraction spot of Li $(1\bar{1}0)$.

In the growth process driven by a low bias of 0.2 V, the growth rate of the right side of Li particle was higher than the left, demonstrating an anticlockwise-rotation growth behavior. The single-crystal Li particle also exposed the low-energy {110}, {112} and {002} planes on different sides. Without mechanical constraint, the freshly generated Li^0 atoms could arrive at these facets through bulk or surface diffusion to form a regular morphology with low free surface energy. The ionic current density during the growth process was estimated to be about 0.02-0.2 $mA\cdot cm^{-2}$, which is obviously lower than that of whisker growth, as the case in Supplementary Fig. 8. This suggests that the low-rate growth of Li facilitates the formation of a morphology with minimized surface area.

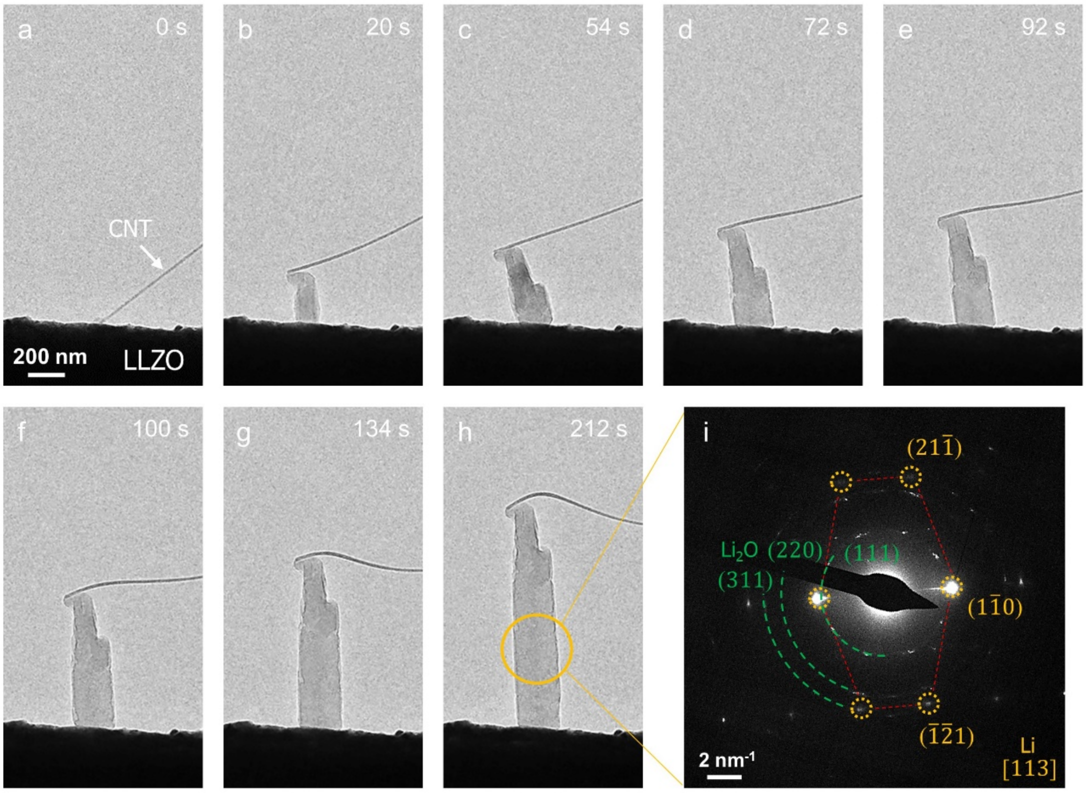

Supplementary Fig. 8 | Free growth of Li metal into a straight whisker. (a-h) Sequential TEM snapshots of the growth of a Li whisker. (i) The SAED pattern of the Li whisker, indicating that the side-wall surface was Li {110} planes with the lowest surface energy.

During the whisker growth, the soft CNT imposed almost no constraint on the growing whisker, whose tip shape always remained unchanged, indicating a root growth mode. The current density for Li whisker growth was estimated to be ~4 mA·cm^{-2}, which is higher than that of the particle growth in Supplementary Fig. 7. This confirms that high-rate growth of Li enables the high-aspect-ratio morphology with increased surface area, where the {110} planes are maximized at the side wall to reduce the overall energy. The rate-dependent morphology for Li growth is in consistence with the recent in situ observation on the growth of Na metal.[16]

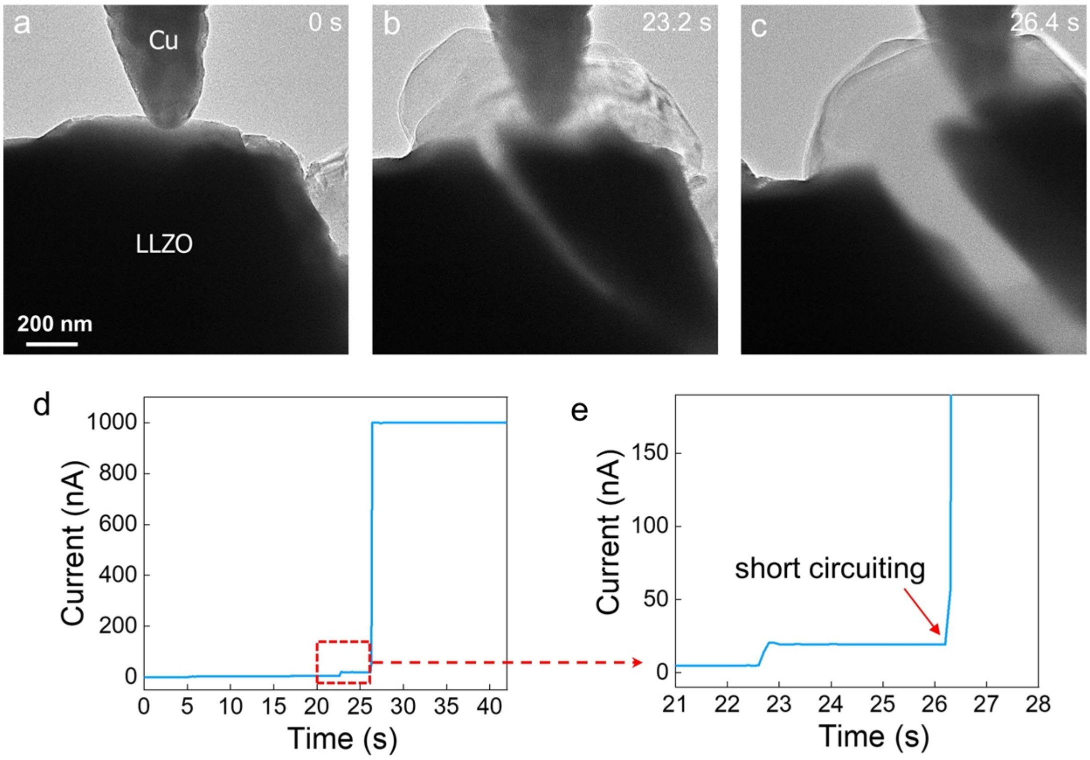

Supplementary Fig. 9 | Crack initiation/propagation and Li penetration upon plating. (a) Cu|LLZO contact made for subsequent Li deposition. (b) During Li plating, a crack was initiated under the accumulated stress. (c) The crack was opened by the growing Li, which later fully penetrate and short circuit the particle. It is interesting to note that the cracking site is a little away from the Cu|LLZO contact point. This suggests that the high stress can be transmitted through the deposited single-crystal Li which can crack the LLZO beneath the Li. (d-e) The measured current curve during the whole process and the enlarged profile of the boxed region at the time from crack initiation to short circuiting (reaches the upper limit of the current range).

It takes a relatively long time (more than 3 s, from~23 s to ~26 s in Supplementary Fig. 9e) for the deposited Li to fully penetrate the LLZO particle to trigger the short circuiting due to the crack opening that increase the amount of Li to fill the crack. This is in sharp contrast to the cases in Supplementary Fig. 10, the short circuiting happened so quickly after cracking in less than 5 ms, attributed to the very narrow gap constrained by the neighboring LLZO particles.

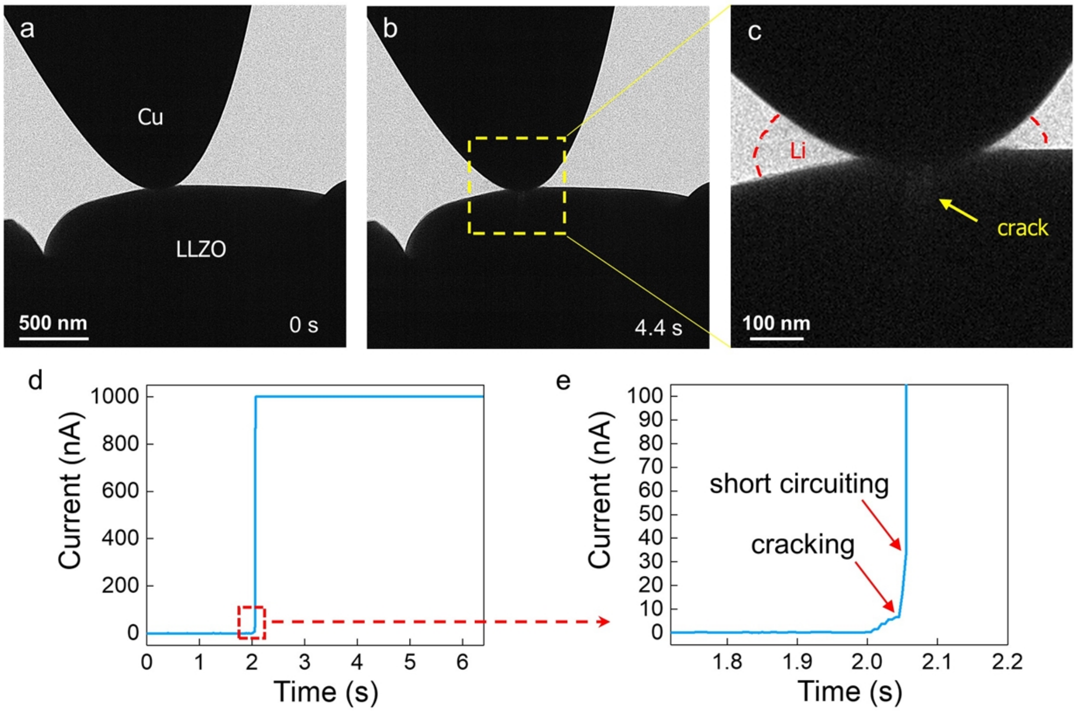

Supplementary Fig. 10 | The fast Li penetration leading to an instant internal short circuiting. (a-b) Upon Li deposition, a crack is generated accompanied with rapid Li filling and penetration through the LLZO particle, triggering the short circuiting. (c) The magnified image of the contact shows a faint contrast of the crack in LLZO particle (marked by the yellow arrowhead) (d) The recorded current curve. (e) The enlarged current profile of the boxed region in (d) corresponding to the crack initiation and short circuiting.

In this case, the LLZO particle in close contact with the neighboring ones was chosen for the study of Li deposition induced cracking of LLZO. Because of the constraints from the neighboring particles, the cracking happened very "quietly". The very weak contrast of the crack indicates that the crack is only slightly opened and the crack planes are not parallel to the e-beam and therefore seemed to be "hidden" (see Supplementary Movie 10 for the dynamic process of cracking). Nonetheless, the crack initiation and Li penetration can be confirmed by the recorded current in Supplementary Fig. 10d, e. Before the sharp increase in current, the relatively gradual increase up to 10 nA should arise from the ionic current by Li deposition, and the 10 nA at cracking is basically at the same level with other cases in i.e. Supplementary Fig. 9. However, the following short circuiting happened very quickly, in about 5 ms (the time resolution of the current data for this curve is 5 ms). This means that the speed of Li penetration can reach at least 0.4 $mm \cdot s^{-1}$ in the narrow gap. It would trigger a very quick short circuiting in a real SSB, and therefore is more detrimental to SEs compared with the crack opening (as shown in Supplementary Fig. 9).

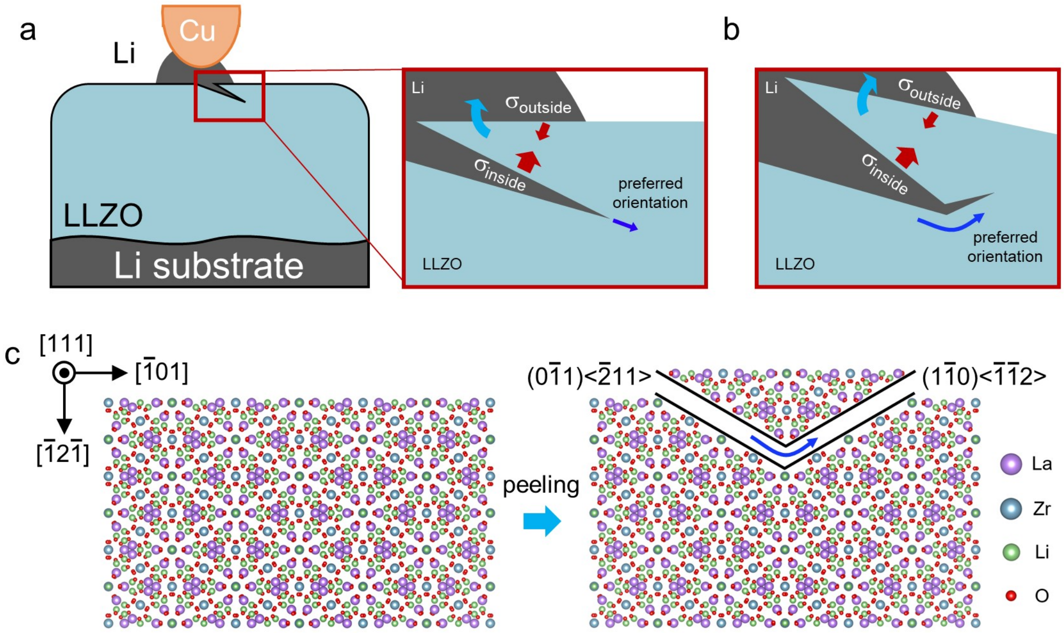

Supplementary Fig. 11 | The schematical of the peeling behavior during high-rate deposition.

As shown in Supplementary Fig. 11a, under high-rate deposition and strong mechanical constraint (from a thick Cu tip), the Li-deposition-induced high stress can initiate the crack along a pre-existed defect or a cleavage plane. If the crack plane forms an acute angle with the particle surface, this crack will be preferentially initiated because of the unbalanced stress, that is, the inside hydrostatic pressure from Li metal is higher than the pressure from the outside surface, as illustrated in the enlarged image in Supplementary Fig. 11a. Moreover, such unbalanced stress can also force the crack to turn its advancing direction. As shown in Supplementary Fig. 11b, during the crack extension in the subsurface, the crack turns its advancing direction towards the particle surface. A possible peeling path is further illustrated in Supplementary Fig. 11c. The {110} planes are one of the low-index planes of the cubic LLZO and prone to be cleaved due to their large interplanar spacing. The crack along $(0\bar{1}1)$ plane is initiated and advances in the <$\bar{2}11$> direction. With the stress build-up in the crack, the crack turns its advancing direction and propagates along the $(1\bar{1}0)$ plane in the <$\bar{1}\bar{1}2$> direction until it arrives at the particle surface. Eventually, the spalled piece is pushed out by the growing Li, as shown in Fig. 3o.

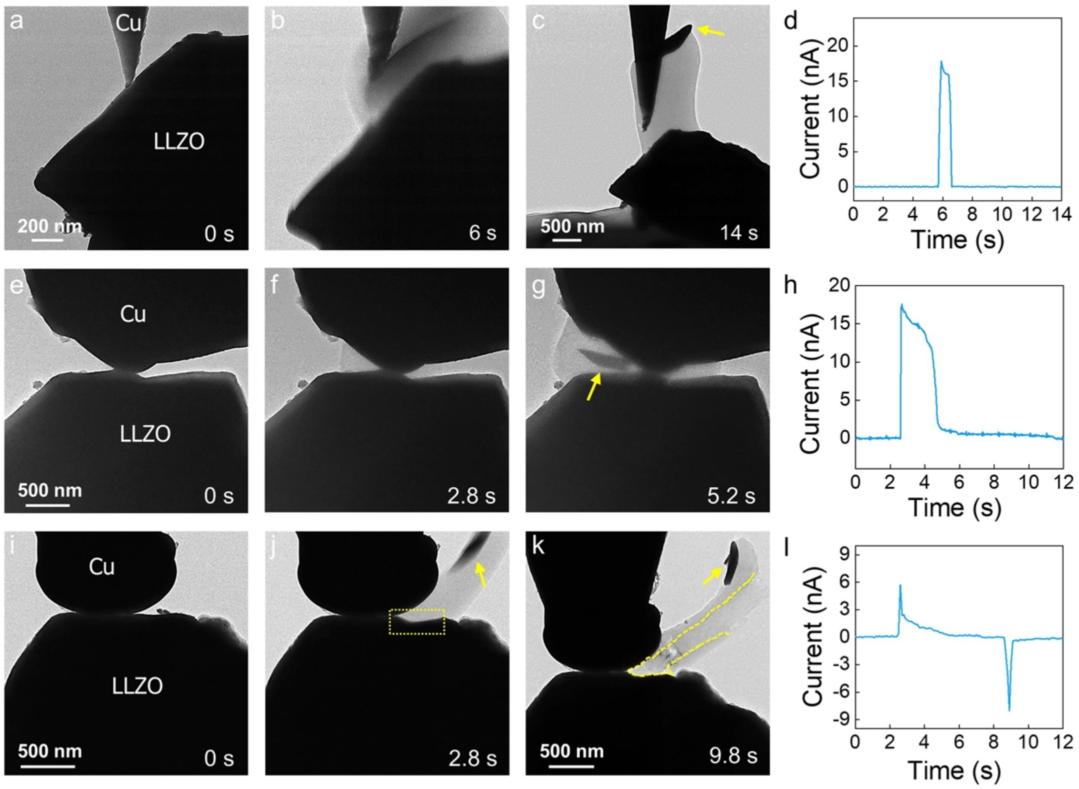

Supplementary Fig. 12 | Surface peeling of LLZO upon Li eruption. (a-c) Li metal grew out rapidly with a fragment of LLZO being peeled off and pushed upward (marked by the yellow arrow). (d) The measured current curve. (e-g) Under the strong constraint condition and high deposition rate, a piece of LLZO was peeled off (marked by the yellow arrowhead). At the eruption moment, the peeling possibly happened at a lower site of the LLZO particle and the spalled piece was therefore not visible (f), which was then pushed out after further Li deposition (g). (h) The measured current during the Li deposition. (i-j) Li eruption carrying the peeled LLZO fragment away from the interface, leaving behind a dent on the LLZO substrate (the peeled piece is marked by the white arrow and the flaw is indicated by the yellow rectangle). (k) Reversing the polarity of the applied voltage leading to the Li stripping from the center of the Li whisker. The empty part after stripping is marked by the yellow dashed line. (l) The two peaks in the current profile correspond to the ionic flux upon plating and stripping, respectively. Note that the Li flux upon stripping did not make a visible damage to the LLZO.

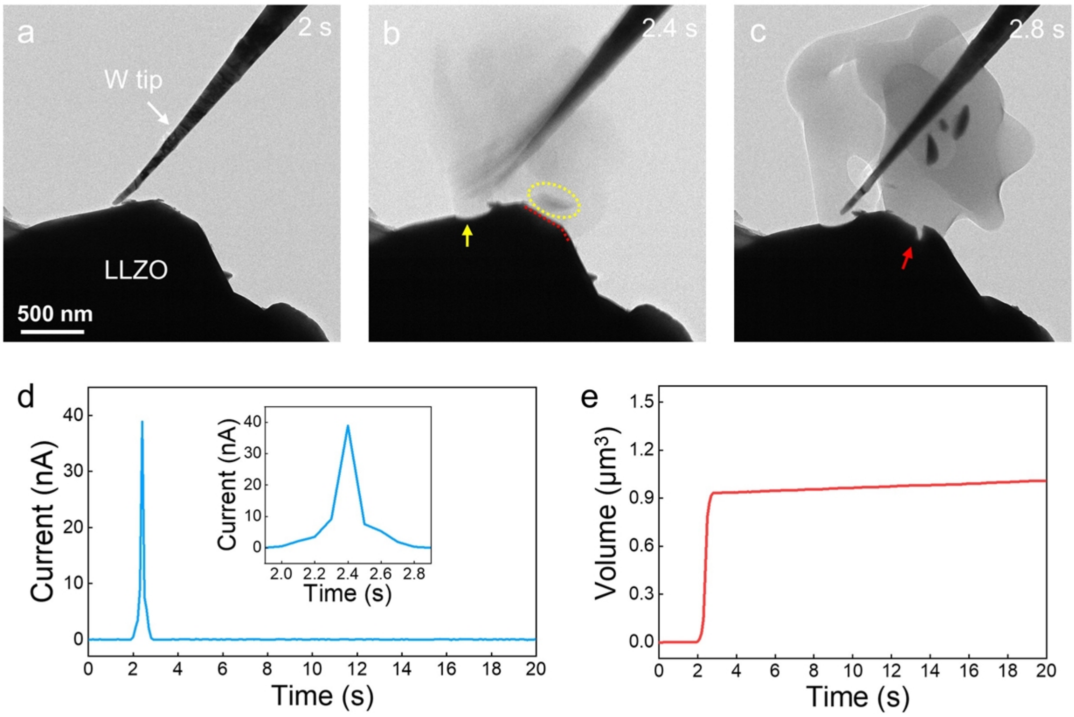

Supplementary Fig. 13 | LLZO damage by both peeling and cracking under a flexible mechanical constraint at high deposition rate. (a) A LLZO particle was made contact with a slender W tip that can be bent so as to offer a variable mechanical constraint. (b) A blurred TEM image captured at the moment of rapid Li deposition at the W|LLZO contact. Such high-rate deposition was triggered by a high applied potential of 3 V. It can still be distinguished that the plated Li metal pushed up the W tip which increased the stack pressure, and a piece of LLZO was peeled off under high stress, leaving behind a dent on the originally flat surface at the contact point (the dent site is marked by the yellow arrow and the spalled piece is circled by the yellow dashed line). Meanwhile, the deposited Li also grew laterally to enlarge the Li|LLZO interface, where the LLZO surface was still flat and intact at this moment, as indicated by the red dashed line. (c) A crack generated on the LLZO surface during the subsequent high-rate deposition (as indicated by the red arrowhead). (d) The measured current curve as a function of deposition time with a notable peak at ~2.4 s. (e) The volume profile of deposited Li metal, in which the sharp increase in volume (~0.93 μm^3, during 2.0-2.8 s, 3.19 $\mu m^3 \cdot s^{-1}$ at 2.4 s) corresponds well to the peeling and cracking event at ~2.4 s.

The LLZO damage in this case demonstrates that the high pressure imposed by the bent and highly stained W tip can be transmitted through deposited Li onto the near LLZO surface. It implies that in a real SSB, the local high stack pressure from the current collector (CC) can be possibly transmitted through the deposited Li metal to the Li|SE interface domains far from the Li|CC contact, and cause Li-deposition-induced stress build up and SE damages.

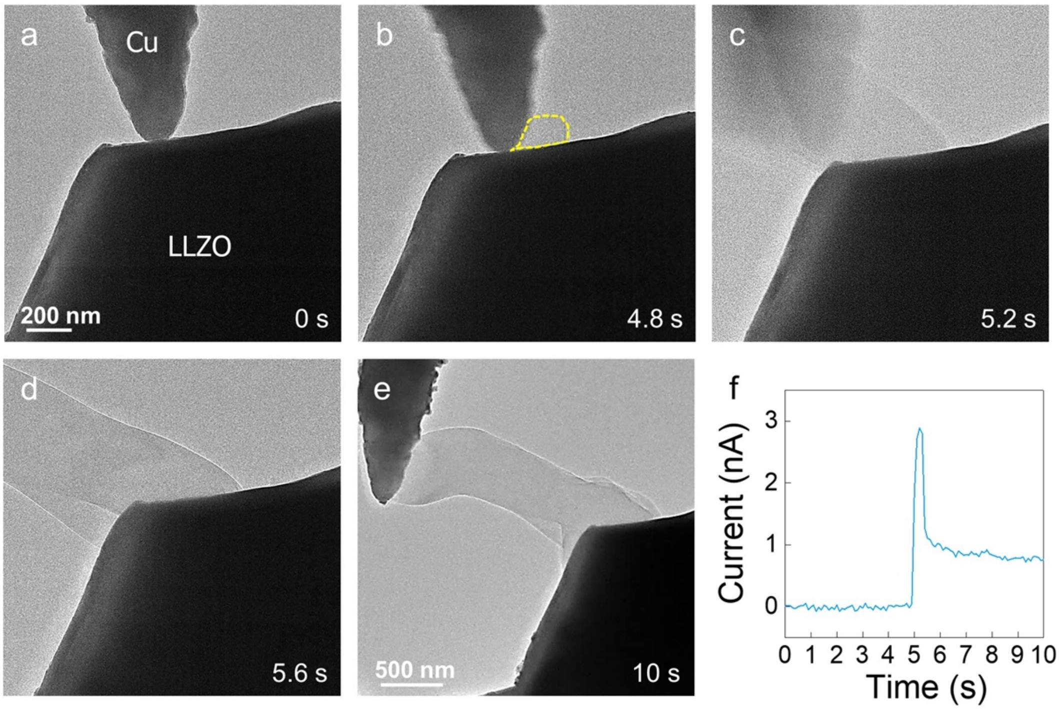

Supplementary Fig. 14 | Li nucleation at one side of the Cu|LLZO contact leading to root growth of Li and deflection of a Cu tip. (a-e) Under a high-rate deposition, Li nucleated at the right side of the Cu tip and its growth pushed the tip leftward rapidly. (f) The current profile shows a spike at 5.2 s, corresponding to a current density of 1590 mA·cm^{-2}.

In spite of the high current density of A·cm^{-2} level, this rapid growth of Li did not cause damage to the LLZO. That is because the pressure imposed by the flexible Cu tip did not reach the growth strength limit of Li, and the root growth can continue to effectively release the stress/mass accumulation at the interface. This suggests that the vertical growth enabled by a floating current collector can be used for the damage-free high-rate Li deposition in SSBs.

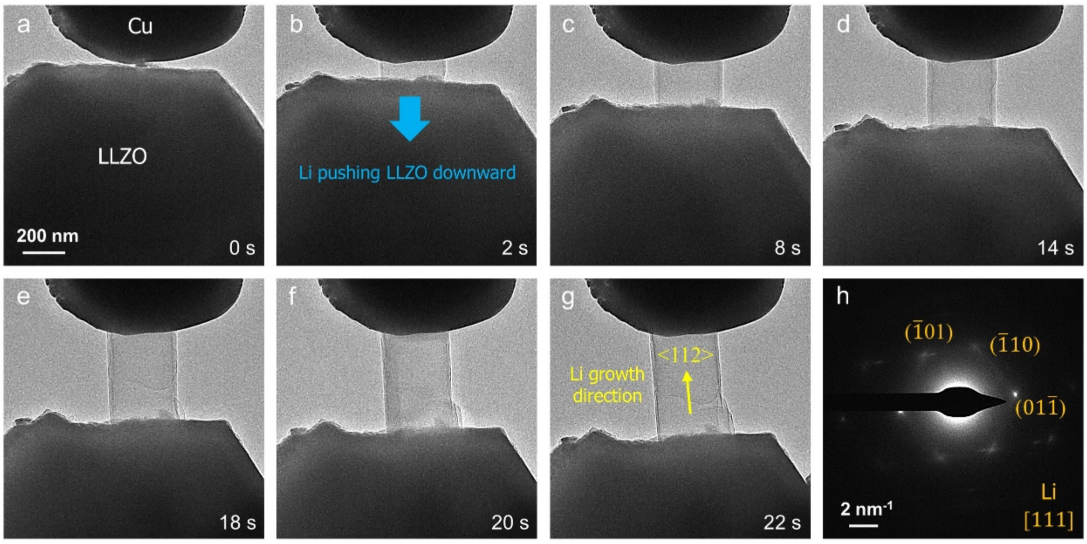

Supplementary Fig. 15 | The vertical Li whisker growth by pushing down the LLZO particle. (a-g) Li growth between a rigid Cu tip and a floating LLZO can push the LLZO downward into the Li metal substrate to release the stress. (h) SAED of the grown Li whisker revealing the growth direction of <112>, which is one of the preferential growth directions of Li whisker.

Similar to the case in Supplementary Fig. 14, the root growth of Li enabled by the relative displacement between CC and LLZO can achieve a high current density without causing damage due to the effective stress/mass release at the Li|SE interface.

5. Chemo-mechanical simulations

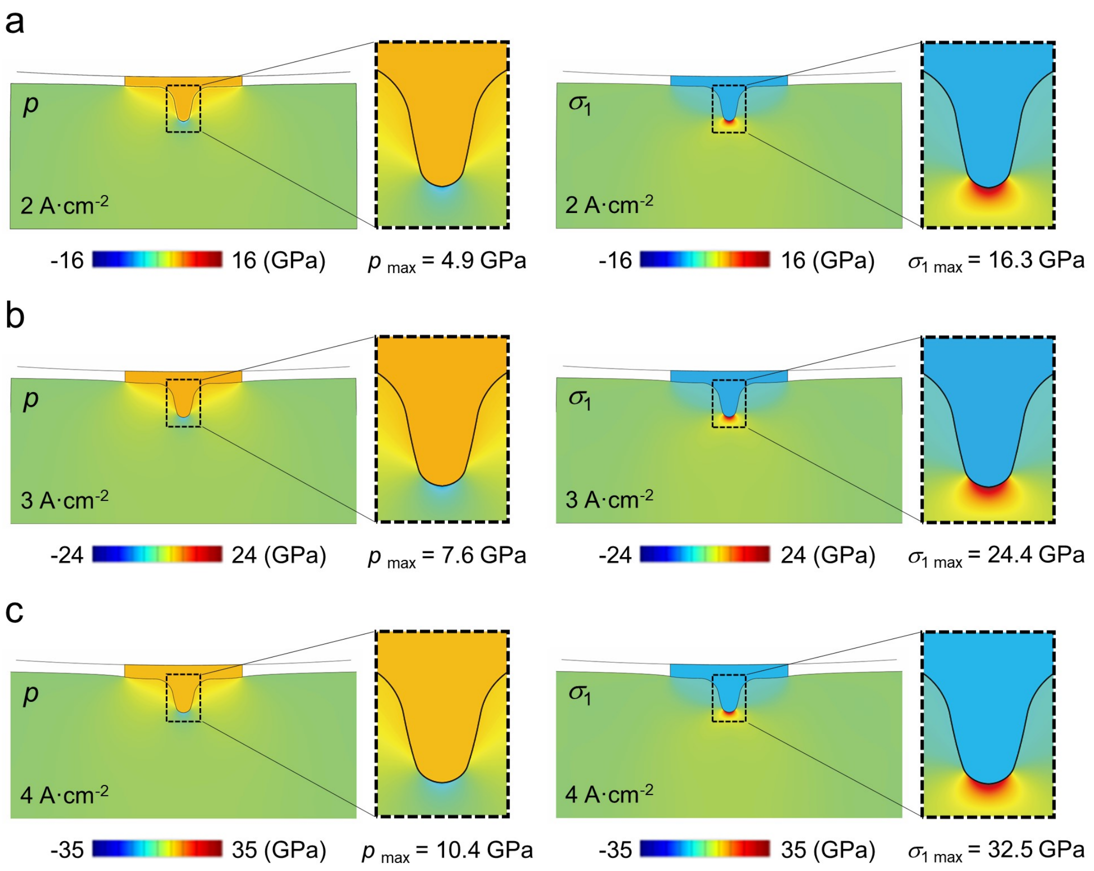

Supplementary Fig. 16 | Chemo-mechanical simulation of the distributions of the hydrostatic pressure (*p*) in Li and the maximum principal stress (σ_1) in LLZO under strong mechanical constraint from CC. The corresponding deposition current densities are: (a) 2 A·cm^{-2}, (b) 3 A·cm^{-2}, and (c) 4 A·cm^{-2}, respectively.

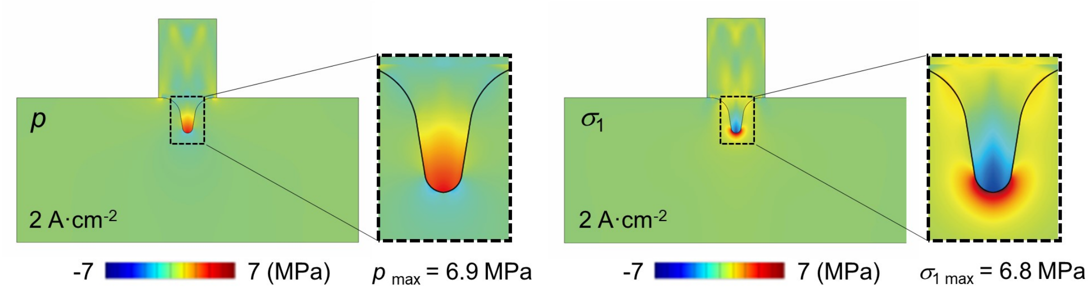

Supplementary Fig. 17 | Chemo-mechanical simulation of the distributions of the hydrostatic pressure (p) in Li and the maximum principal stress (σ_1) in LLZO under no top mechanical constraint. The deposition current density is 2 A·cm^{-2}. This case is equivalent to the fast whisker growth in Fig.4g. The generated p and σ_1 below 10 MPa, and would not cause any damage to LLZO, as observed in Fig. 4g.

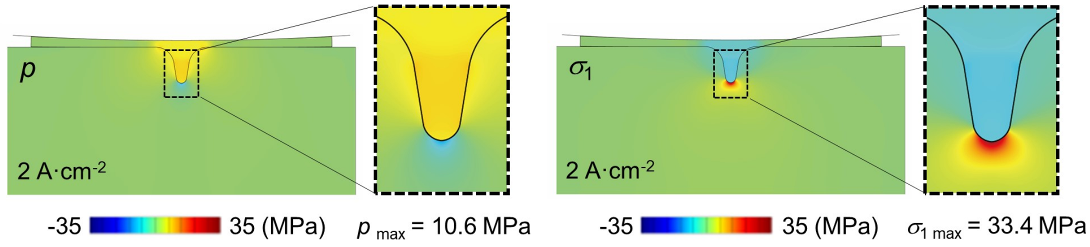

Supplementary Fig. 18 | Chemo-mechanical simulation of the distributions of the hydrostatic pressure (p) in Li and the maximum principal stress (σ_1) in LLZO under high temperature of 468 K. The deposition current density is 2 A·cm^{-2}. The high temperature can significantly promote Li^0 diffusivity and the transition in viscosity of Li, and the rapidly deposited Li can be efficiently transported to the nearby gap space, thus avoiding the pressure accumulation at the interface.

6. Supplementary Movies

Supplementary Movie 1

In situ TEM observation of the lateral growth of Li metal at low deposition rate under strong mechanical constraint (Fig. 1). (Displayed with 8× speed of a real time process)

Supplementary Movie 2

In situ TEM observation of the Li deposition under a variable mechanical constraint imposed by a slender W tip that forces the growing Li to transit from vertical growth to lateral expansion (Fig. 2). (Displayed with 16× speed)

Supplementary Movie 3

In situ TEM observation of the crack initiation and opening and Li propagation in LLZO under high deposition rate and strong mechanical constraint (Fig. 3a-d). (Displayed with 1× speed)

Supplementary Movie 4

In situ TEM observation of the initiation of a narrow crack and Li penetration in a LLZO particle in tight contact with the neighboring particles (Fig. 3i, j). (Displayed with 1× speed)

Supplementary Movie 5

In situ TEM observation of the Li eruption to peel off the LLZO under strong mechanical constraint, leading to two fragments spalled off (Fig. 3m-o). (Displayed with 1× speed)

Supplementary Movie 6

In situ TEM observation of the fast Li whisker growth with a slender carbon nanotube as the current collector that exerts negligible constraint ((Fig. 4g). (Displayed with 1× speed)

Supplementary Movie 7

In situ TEM observation of the Li fast plating inside an amorphous carbon nanotube between the current collector and LLZO via Li^+ (or Li^0) transport along the nanotube (Fig. 4i). (Displayed with 1× speed)

Supplementary Movie 8

In situ TEM observation of the free growth of Li metal into a single-crystal faceted particle on LLZO induced by a CNT (Supplementary Fig. 7). (Displayed with 4× speed)

Supplementary Movie 9

In situ TEM observation of the CNT-induced Li whisker growth on LLZO with a relatively low rate (Supplementary Fig. 8). (Displayed with 8× speed)

Supplementary Movie 10

In situ TEM observation of the cracking of a LLZO particle that is constrained from opening by the neighboring particles, leading to superfast Li penetration (Supplementary Fig. 10). (Displayed with 1× speed)

Supplementary Movie 11
In situ TEM observation of the surface peeling of LLZO by fast Li deposition (Supplementary Fig. 12a-c). (Displayed with 1× speed)

Supplementary Movie 12
In situ TEM observation of the surface peeling of LLZO by fast Li deposition (Supplementary Fig. 12e-g). (Displayed with 1× speed)

Supplementary Movie 13
In situ TEM observation of the surface peeling of LLZO by fast Li deposition (Supplementary Fig. 12i-k). (Displayed with 1× speed)

Supplementary Movie 14
In situ TEM observation of the peeling and cracking of LLZO upon Li eruption under a variable constraint imposed by a slender W tip (Supplementary Fig. 13). (Displayed with 1× speed)

Supplementary Movie 15
In situ TEM observation of the fast Li lateral growth enabled by the deflection of a Cu tip (Supplementary Fig. 14). (Displayed with 1× speed)

Supplementary Movie 16
In situ TEM observation of the vertical growth of a Li whisker by pushing down the LLZO particle (Supplementary Fig. 15). (Displayed with 1× speed)

Supplementary Movie 17
A movie showing the simulated stress evolution at the Li|LLZO interface at low local current density of 4 $mA\cdot cm^{-2}$ under strong top mechanical constraint (Fig. 4b, c).

Supplementary Movie 18
A movie showing the simulated stress evolution at the Li|LLZO interface at different high current densities under strong mechanical constraint (Supplementary Fig. 16).

Supplementary Movie 19
A movie showing the simulated stress evolution at the Li|LLZO interface, corresponding to the case of rapid Li whisker growth on LLZO without top mechanical constraint (Supplementary Fig. 17).

Supplementary Movie 20
A movie showing the simulated stress evolution at the Li|LLZO interface at high current density of 2 $A\cdot cm^{-2}$ under strong top mechanical constraint and high temperature of 468 K (Supplementary Fig. 18).

Supplemental Reference

1. Yu S. et al. Elastic properties of the solid electrolyte $Li_7La_3Zr_2O_{12}$ (LLZO). *Chem. Mat.* **28**, 197-206 (2016).
2. Narayan S. & Anand L. On modeling the detrimental effects of inhomogeneous plating-and-stripping at a lithium-metal/solid-electrolyte interface in a solid-state-battery. *J. Electrochem. Soc.* **167**, 040525 (2020).
3. Xiong L. et al. A solvent molecule driven pure PEDOT:PSS actuator. *Macromol. Mater. Eng.* **305**, 2000327 (2020).
4. Brown S. B., Kim K. H. & Anand L. An internal variable constitutive model for hot working of metals. *Int. J. Plast.* **5**, 95-130 (1989).
5. LePage W. S. et al. Lithium mechanics: roles of strain rate and temperature and implications for lithium metal batteries. *J. Electrochem. Soc.* **166**, A89-A97 (2019).
6. Sharafi A. et al. Surface chemistry mechanism of ultra-Low interfacial resistance in the solid-state electrolyte $Li_7La_3Zr_2O_{12}$. *Chem. Mat.* **29**, 7961-7968 (2017).
7. Zheng H. et al. Intrinsic Lithiophilicity of Li–Garnet Electrolytes Enabling High-Rate Lithium Cycling. *Adv. Funct. Mater.* **30**, 1906189 (2019).
8. Gao B., Jalem R. & Tateyama Y. Surface-dependent stability of the interface between garnet $Li_7La_3Zr_2O_{12}$ and the Li metal in the all-solid-state battery from first-principles calculations. *ACS Appl. Mater. Interfaces* **12**, 16350-16358 (2020).
9. Wang M. & Sakamoto J. Correlating the interface resistance and surface adhesion of the Li metal-solid electrolyte interface. *J. Power Sources* **377**, 7-11 (2018).
10. Krauskopf T., Hartmann H., Zeier W. G. & Janek J. Toward a Fundamental Understanding of the Lithium Metal Anode in Solid-State Batteries-An Electrochemo-Mechanical Study on the Garnet-Type Solid Electrolyte $Li_{6.25}Al_{0.25}La_3Zr_2O_{12}$. *ACS Appl. Mater. Interfaces* **11**, 14463-14477 (2019).
11. Cockayne B. Strong solids. *Phys. Bull.* **18**, 25-25 (1967).
12. MacMillan N. H. The theoretical strength of solids. *J. Mater. Sci.* **7**, 239-254 (1972).
13. He Y. et al. Origin of lithium whisker formation and growth under stress. *Nat. Nanotechnol.* **14**, 1042-1047 (2019).
14. Zhang L. et al. Lithium whisker growth and stress generation in an in situ atomic force microscope-environmental transmission electron microscope set-up. *Nat. Nanotechnol.* **15**, 94-98 (2020).
15. Wang M. S., Peng L. M., Wang J. Y. & Chen Q. Shaping carbon nanotubes and the effects on their electrical and mechanical properties. *Adv. Funct. Mater.* **16**, 1462-1468 (2006).
16. Liu Q. N. et al. In situ observation of sodium dendrite growth and concurrent mechanical property measurements using an environmental transmission electron microscopy-atomic force microscopy (ETEM-AFM) platform. *ACS Energy Lett.* **5**, 2546-2559 (2020).
