# Supplementary material for: Visualizing the failure of solid electrolyte under GPa-level interface stress induced by lithium eruption
Source: Nat Commun. 2022 Aug 27;13:5050. doi: 10.1038/s41467-022-32732-z (PMC9420139; doi:10.1038/s41467-022-32732-z)
Supplement: Supplementary file 2 — Description of Additional Supplementary Files [file 41467_2022_32732_MOESM2_ESM.pdf]

Description of Additional Supplementary Files

Supplementary Movie 1
In situ TEM observation of the lateral growth of Li metal at low deposition rate under strong mechanical constraint (Fig. 1). (Displayed with 8× speed of a real time process)

Supplementary Movie 2
In situ TEM observation of the Li deposition under a variable mechanical constraint imposed by a slender W tip that forces the growing Li to transit from vertical growth to lateral expansion (Fig. 2). (Displayed with 16× speed)

Supplementary Movie 3
In situ TEM observation of the crack initiation and opening and Li propagation in LLZO under high deposition rate and strong mechanical constraint (Fig. 3a-d). (Displayed with 1× speed)

Supplementary Movie 4
In situ TEM observation of the initiation of a narrow crack and Li penetration in a LLZO particle in tight contact with the neighboring particles (Fig. 3i, j). (Displayed with 1× speed)

Supplementary Movie 5
In situ TEM observation of the Li eruption to peel off the LLZO under strong mechanical constraint, leading to two fragments spalled off (Fig. 3m-o). (Displayed with 1× speed)

Supplementary Movie 6
In situ TEM observation of the fast Li whisker growth with a slender carbon nanotube as the current collector that exerts negligible constraint ((Fig. 4g). (Displayed with 1× speed)

Supplementary Movie 7
In situ TEM observation of the Li fast plating inside an amorphous carbon nanotube between the current collector and LLZO via Li^+ (or Li^0) transport along the nanotube (Fig. 4i). (Displayed with 1× speed)

Supplementary Movie 8
In situ TEM observation of the free growth of Li metal into a single-crystal faceted particle on LLZO induced by a CNT (Supplementary Fig. 7). (Displayed with 4× speed)

Supplementary Movie 9
In situ TEM observation of the CNT-induced Li whisker growth on LLZO with a relatively low rate (Supplementary Fig. 8). (Displayed with 8× speed)

Supplementary Movie 10
In situ TEM observation of the cracking of a LLZO particle that is constrained from opening by the neighboring particles, leading to superfast Li penetration (Supplementary Fig. 10). (Displayed with 1× speed)

Supplementary Movie 11
In situ TEM observation of the surface peeling of LLZO by fast Li deposition (Supplementary Fig. 12a-c). (Displayed with 1× speed)

Supplementary Movie 12
In situ TEM observation of the surface peeling of LLZO by fast Li deposition (Supplementary Fig. 12e-g). (Displayed with 1× speed)

Supplementary Movie 13
In situ TEM observation of the surface peeling of LLZO by fast Li deposition (Supplementary Fig. 12i-k). (Displayed with 1× speed)

Supplementary Movie 14
In situ TEM observation of the peeling and cracking of LLZO upon Li eruption under a variable constraint imposed by a slender W tip (Supplementary Fig. 13). (Displayed with 1× speed)

Supplementary Movie 15
In situ TEM observation of the fast Li lateral growth enabled by the deflection of a Cu tip (Supplementary Fig. 14). (Displayed with 1× speed)

Supplementary Movie 16
In situ TEM observation of the vertical growth of a Li whisker by pushing down the LLZO particle (Supplementary Fig. 15). (Displayed with 1× speed)

Supplementary Movie 17
A movie showing the simulated stress evolution at the Li|LLZO interface at low local current density of 4 $mA\cdot cm^{-2}$ under strong top mechanical constraint (Fig. 4b, c).

Supplementary Movie 18
A movie showing the simulated stress evolution at the Li|LLZO interface at different high current densities under strong mechanical constraint (Supplementary Fig. 16).

Supplementary Movie 19
A movie showing the simulated stress evolution at the Li|LLZO interface, corresponding to the case of rapid Li whisker growth on LLZO without top mechanical constraint (Supplementary Fig. 17).

Supplementary Movie 20
A movie showing the simulated stress evolution at the Li|LLZO interface at high current density of 2 $A\cdot cm^{-2}$ under strong top mechanical constraint and high temperature of 468 K (Supplementary Fig. 18).
